# Efficacy thresholds and target populations for antiviral COVID-19 treatments to save lives and costs: a modelling study

**DOI:** 10.1016/j.eclinm.2024.102683

**Published:** 2024-06-21

**Authors:** Epke A. Le Rutte, Andrew J. Shattock, Inês Marcelino, Sophie Goldenberg, Melissa A. Penny

**Affiliations:** a Department of Epidemiology and Public Health, Swiss Tropical and Public Health Institute, Allschwil, Switzerland; b University of Basel, Basel, Switzerland; c Telethon Kids Institute, Nedlands, WA, Australia; d Centre for Child Health Research, University of Western Australia, Crawley, WA, Australia; e Institute for Risk Assessment Sciences, Utrecht University, Utrecht, Netherlands; f Department of Quantitative Veterinary Epidemiology, Wageningen University & Research, Wageningen, Netherlands; g Department of Medicine, Health, and Society, Vanderbilt University, Nashville, USA

**Keywords:** SARS-CoV-2, Treatment, Transmission modelling, Economic and public health impact

## Abstract

**Background:**

In 2023 severe acute respiratory syndrome coronavirus 2 (SARS-CoV-2) was declared endemic, yet hospital admissions have persisted and risen within populations at high and moderate risk of developing severe disease, which include those of older age, and those with co-morbidities. Antiviral treatments, currently only available for high-risk individuals, play an important role in preventing severe disease and hospitalisation within this subpopulation. Here, we further explore the public health and economic benefits of extending target populations for treatment, and assess efficacy thresholds for a treatment strategy to be cost-saving.

**Methods:**

We adapted an individual-based transmission model of SARS-CoV-2, OpenCOVID, which was calibrated and validated to 2020–2023 Swiss, European, and Northern Hemisphere epidemiological data. We used the model to estimate hospitalisations and overall costs for preventatively treating three risk groups for a full range of treatment efficacies and coverages with, besides vaccination and hospital treatments, no other interventions in place. We further calculated efficacy thresholds for strategies to be cost-saving. A global sensitivity analysis was conducted to test the sensitivity of all outcomes for a wide range of treatment properties, emerging variant properties, and vaccination coverages.

**Findings:**

In a high vaccination coverage setting, we found that a high efficacy antiviral treatment given to all those at high-risk could reduce hospitalisations by up to 40%. When expanding treatment coverage to also include all those at moderate-risk, an additional 50% of hospitalisations could be averted. Targeting both high-risk and moderate-risk groups was found to be cost-saving for a treatment efficacy greater than ∼40%. This threshold was found to be robust regardless of vaccination coverage and emerging variant properties, but highly sensitive to treatment costs.

**Interpretation:**

For a sufficiently efficacious antiviral treatment, expanding the target population to include both high-risk and moderate-risk groups should be considered. Equitable treatment costs are found crucial in achieving the best possible public health and health economic outcomes.

**Funding:**

Botnar Research Centre for Child Health (DZX2165 to MAP), the 10.13039/100000001Swiss National Science Foundation Professorship of MAP (P00P3_203450) and 10.13039/100000001Swiss National Science Foundation NFP 78 Covid-19 2020 (4079P0_198428 to MAP).

**Research in context**

**Evidence before this study**

We use an established, peer-reviewed, and fully open access, individual-based transmission model of severe acute respiratory syndrome coronavirus 2 (SARS-CoV-2), OpenCovid, which has been fitted and validated against epidemiological data from northern hemisphere settings, and has been applied to multiple research and policy-related questions. For this study, the model has been extended with novel features to include the latest available published data and scientific findings between January and August 2023, for which the PubMed search terms included; antiviral treatment, efficacy, SARS-CoV-2, COVID-19, immunity, and mathematical modelling. For the simulated scenarios we used publicly available information from a range of countries regarding their current antiviral treatment target population definitions and treatment and hospital associated costs.

**Added value of this study**

We provide the first full quantitative guidance on the potential health and economic impact of new antiviral treatments against COVID-19 hospitalisations and intensive care unit admissions for a range of future scenarios concerning vaccination coverage rates and viral variants. This evidence supports discussion around requirements for novel treatment development, their use cases, and the drivers behind their potential success. It importantly also highlights under which circumstances certain antiviral treatment scenarios are not cost-saving.

**Implications of all the available evidence**

Our novel approach and outcomes are of key interest to the scientific community, product developers, and policy makers. When future antiviral treatments are developed and clinical trials provide first efficacy values, these can be checked within the full range of efficacy values simulated by us. The plots can inform under which circumstances and target population that particular treatment could potentially be cost-saving, and which underlying factors drive that outcome.

## Introduction

In 2022 and 2023 multiple countries around the world continued to see increasing waves of severe acute respiratory syndrome coronavirus 2 (SARS-CoV-2) infection rates and hospitalisations.^1^ This was partially caused by waning naturally-acquired and vaccine-induced immunity^2^, ^3^, ^4^, ^5^ and diminishing vaccine booster uptake rates.^6^ Hospital capacities may continue to be threatened by the emergence of novel (immune evading and potentially more severe) variants^7^ and parallel influenza and respiratory syncytial virus (RSV) waves.^8^ Therefore, continued and novel COVID-19 pharmaceuticals are necessary to continue to mitigate pressure on public health and healthcare systems.^9^

In late 2021 and early 2022, the World Health Organization (WHO), United States Food and Drug Administration (FDA), and European Medicine Agency (EMA) issued emergency use authorization for two antiviral SARS-CoV-2 treatments: Paxlovid (nirmatrelvir and ritonavir) and Lagevrio (molnupiravir). These treatments prevent viral replication and reduce the risk of hospitalisation and death from COVID-19.^10^, ^11^, ^12^, ^13^ Within their current use case they are available for individuals who test positive for SARS-CoV-2 and are at high risk of developing severe COVID-19 disease, specifically those of old age and with comorbidities.^13^^,^^14^ Lower-risk individuals have been excluded from use of these treatments, given the lack of statistically significant efficacy^15^ and negative side effects, namely, rebound COVID-19 symptoms.^16^

Whilst these antiviral SARS-CoV-2 treatments were initially effective at reducing the risk of developing severe COVID-19 symptoms and hospitalisation when taken within days of developing symptoms, they were trialled before wide-scale vaccine use and development of naturally acquired immunity.^17^ Outside of trials, these treatments have presented a wide range of efficacies under varying circumstances.^18^^,^^19^ Treatment efficacy requirements and the target populations who will benefit the most from (partially) efficacious treatments remain largely unknown, especially in settings where novel variants could become more severe and vaccination coverage rates may vary. Therefore, clinical trials of (new) antiviral treatments will be required to demonstrate the efficacy at the patient level in diverse circumstances. However, while the efficacies of a new drug in clinical trials will quantify the benefits at an individual level, the benefit at population level and associated health cost savings requires analysis beyond a clinical study, for which transmission modelling is well suited. Modelling outcomes can provide initial evidence to understand the drivers behind the treatment's health and economic impact and provide insight into required efficacies for a range of target populations, varying coverage rates, and future variant scenarios.

This study primarily aims to understand the dynamics underlaying the health and economic impact of antiviral treatments for different target patient populations under a wide range of hypothetical future transmission and variant scenarios. Adapting and using a SARS-CoV-2 transmission model (OpenCOVID^20^), we address these questions for an archetypal Northern Hemisphere transmission setting. We use an individual-based model structure to capture heterogeneities behind each individual's risk to develop severe disease, which is what the antiviral treatments aim to prevent.

Firstly, we analyse the public health impact of expanding treatment coverage from only the high-risk population to include those at moderate and low risk of developing severe disease for a full range of antiviral treatment efficacies. Secondly, we identify the antiviral treatment efficacy threshold at which each use case becomes cost-saving.

For both the public health and economic impact we undertook an extensive sensitivity analysis to explore how epidemiological factors influence these outcomes, including the emergence of more severe and immune-evading variants, a range of vaccination coverage rates, and antiviral treatment costs.

Herewith, we provide the first full quantitative guidance on the potential health and economic impact of new antiviral treatments against hospitalisations and intensive care unit (ICU) admissions for a range of future scenarios concerning vaccination and variants. This evidence supports discussion around requirements for novel treatment development and use cases.

## Methods

### OpenCOVID

We use OpenCOVID, an established individual-based model, which captures the transmission and infection dynamics of SARS-CoV-2, host immunity, and disease progression. This transmission model has been used in previous context to analyse the impact of non-pharmaceutical interventions, vaccine strategies, and novel variants.^20^, ^21^, ^22^ A key feature of this individual-based model is its fine granularity in capturing each individual's immunity by keeping record of past vaccinations and past infections. The individual's immunity levels as well as the persons age and any potential comorbidities influence the risk of new infections and the level of any future disease severity and thus their potential need for hospitalisation. With antiviral treatments preventing severe disease in the individual, capturing this level of individual heterogeneity with an individual-based model is particularly important. Additionally, once an individual is treated and fully recovered, their viral load is cleared and the individual is no longer infectious, and thus they no longer transmit the virus, influencing the population transmission dynamics for which a dynamic individual-based model is specifically suitable. For this study, we adapted OpenCOVID (version 4.0) to simulate antiviral treatments, and further updated waning immunity patterns regarding hybrid vaccine dose and exposure response relationships. We further incorporated health economic costs associated with treatment and hospital care. We calibrated key model parameters to arrive at a pre-defined effective reproduction number (Re of 0.9) at the start of model simulations (spring 2023) to represent an archetypal Northern Hemisphere transmission setting (with seasonal patterns). Below we describe the parameters that are essential to this study, additional model details and parameters (e.g. covering immunity, viral load, viral variants, seasonality patterns) are described in the Supplementary Information as well as in previous OpenCOVID application publications.^20^, ^21^, ^22^ Complete model code and all configuration files required to reproduce this analysis are open access and available from the OpenCOVID git-repository: github.com/SwissTPH/OpenCOVID/tree/manuscript_treatment.^23^

### Antiviral treatment

In the model, the effect of an individual receiving antiviral treatment is binary; the individual will either fully recover following treatment, or there will be no effect and disease progression may proceed. Within the model and to align to the clinical trial outcomes, full recovery means that the viral load is cleared and the individual is no longer infected, infectious, at risk of severe disease and thus also no longer requires hospitalisation. The probability of these outcomes depends entirely on treatment efficacy; a treatment efficacy of 80% means that 80% of those who receive treatment will fully recover following treatment and that in 20% of individuals the treatment will have no effect.

Other adjustable treatment associated parameters include: treatment coverage per risk group, treatment adherence, start date of treatment availability, and delay to treatment effect. Everyone who gets infected in the model is assumed to be diagnosed and eligible for treatment. We assumed a 2-day delay between the start of symptoms and being diagnosed, followed by a 3-day delay to take the treatment. We further assumed universal treatment adherence for those eligible.

### Target populations

We define three target sub-populations. Firstly, those at high risk of getting severe COVID-19 disease, which we defined as individuals >65-years old and those of all ages with co-morbidities, which is about 25% of the total population for our archetypal setting.^24^ Secondly, those at moderate risk of developing severe symptoms are defined as 40–65-years old and without co-morbidities. Thirdly, those at low risk of getting severe disease are defined as 18–40-year olds without co-morbidities.

### Health costs

Intervention costs consist of the antiviral treatment unit cost (one full treatment regimen) multiplied by the number of people that receive the treatment. The cost of each antiviral dose has been based on existing antiviral costs of around €500^25^ and was ranged for the sensitivity analysis. In this study we express an individual intervention cost as a percentage of the average healthcare costs for one full COVID-19 hospital admission.

Healthcare costs are therefore representative too and were calculated as a single cost per person for one full hospitalisation including a potential ICU admission.^26^, ^27^, ^28^ Per person we assumed a representative total cost of €10,000 per hospitalisation and €20,000 per stay in ICU derived from several European countries, which is detailed in the Supplementary Information.^26^ For these representative costs of €10,000, the current cost of one treatment regimen (€500) is about 5% of that of a hospital stay.

Adding the intervention and healthcare costs together leads to the overall costs concerned.

### Statistics

#### Scenarios

Alongside a baseline ‘no antiviral treatment’ scenario, we first simulated two example treatment products (with 30% and 90% treatment efficacy). This was done for the high-risk population (the current default strategy) and also for two alternative strategies by also including those of moderate-risk and including those of moderate and low-risk, all with 100% treatment coverage. Secondly, we simulated a full factorial range of scenarios, for treatment efficacies of 0–100% and target population coverages of 0–100%, for both public health and cost-related outcomes. Thirdly, we identified the efficacy threshold for which each treatment strategy becomes cost-saving. All scenarios are simulated for two years into the future. Each scenario is repeated 50 times, each time using a different random number generator starting seed, to capture stochastic uncertainty.

Uncertainty bounds are included to represent approximate 95% percentile range (2.5%–97.5%) from the temporal distribution from these 50 simulations.

#### Sensitivity analyses

We conducted a sensitivity analysis to assess how sensitive model outcomes are to various model parameters. We first did a global sensitivity analysis on costs (intervention costs, healthcare costs, and combined overall costs), followed by a sensitivity analysis on the cost-saving efficacy threshold for each risk group. The parameters included were: 1) future variant severity, 2) future variant immune evading capacity, 3) primary vaccination coverage, 4) booster coverage, 5) treatment costs, 6) treatment coverage, and 7) treatment efficacy. An overview of the parameters’ default values and their sensitivity analysis ranges is presented in Table 1. Further details of the global sensitivity analysis are described in the Supplementary methods section of the Supplementary Information.

**Table 1:** Parameter values and ranges.

Parameter	Reference value	Range for global sensitivity analysis
Treatment cost	€500 per regimen	€0–1000
Primary vaccine coverage	80% (for all risk-groups)	0–100%
Vaccine booster coverage	50% (for all risk-groups)	0–100%
Variant immune evading	5% (relative to the previous variant)	0–100%
Variant severity	Equal to the previous variant	−50% —+*50%*
Treatment coverage	N/A (ranged throughout the analysis)	0–100%
Treatment efficacy	N/A (ranged throughout the analysis)	0–100%

### Ethics

No ethical approval was required due to the nature of this study; a simulation modelling study of the transmission dynamics of SARS-CoV-2.

### Role of funding source

The Funders had no role in the study design, data collection, data analyses, interpretation, or writing of report.

## Results

### Public health outcomes

**Fig. 1:**
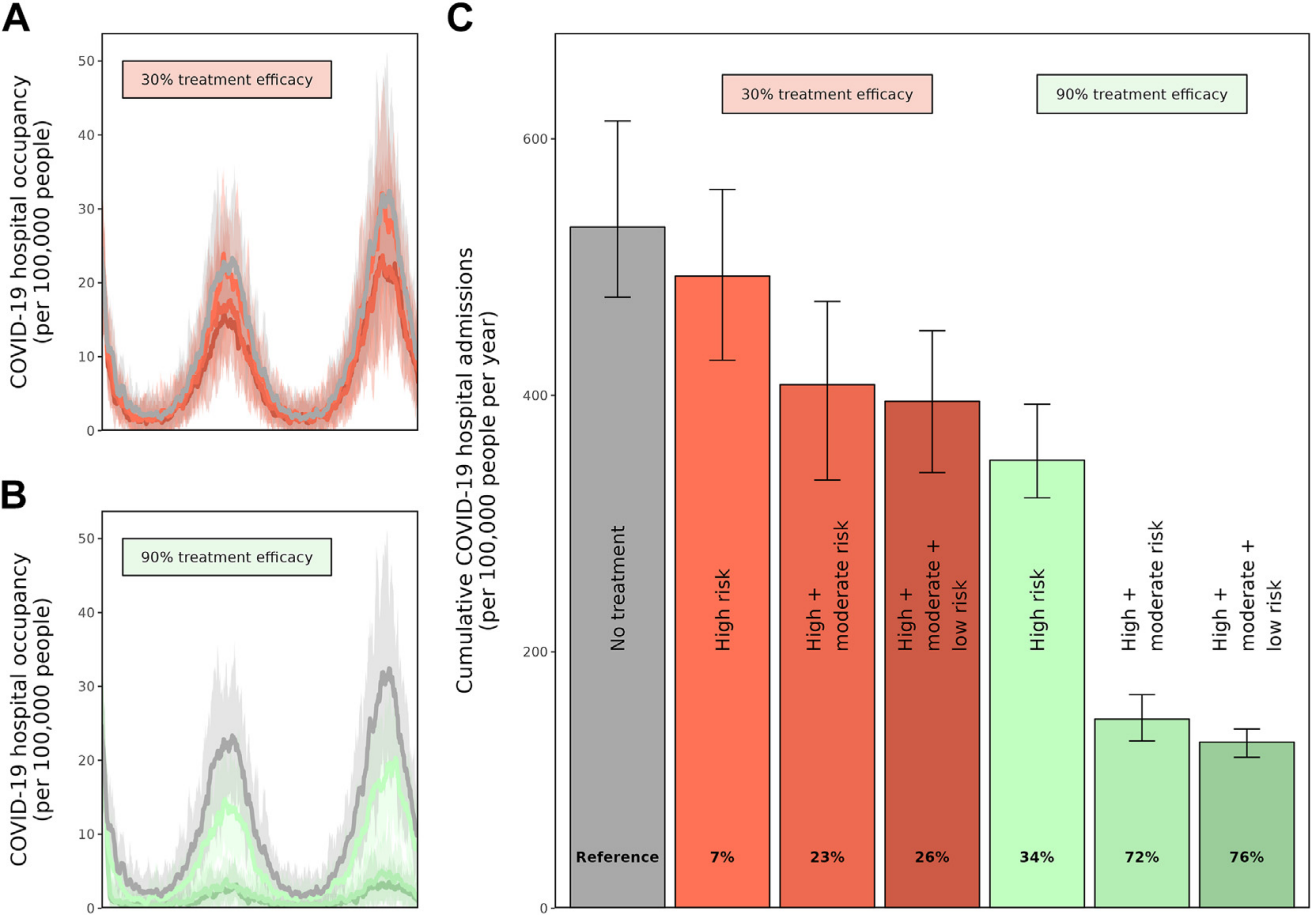
**Two-year temporal pattern (A, B) and cumulative values (C) of COVID-19 hospital admissions per 100,000 population for seven different antiviral treatment scenarios**. The uncertainty bounds represent 95% prediction intervals. The calculated impact (as percentage hospital admissions averted) are shown in the cumulative bar chart (C). In the baseline scenario (grey curve) there are no antiviral treatments available. Two types of antiviral treatments that currently exist are simulated, with a low treatment efficacy of 30% (orange shades) and high efficacy of 90% (green shades). Simulations in which only those at high risk of developing severe COVID-19 disease are treated (the current treatment target population, default) are presented with lighter shades of colour. Simulations in which antiviral treatment is extended to include also those at moderate and low risk of developing severe COVID-19 disease are represented by the darker shades of colour. Novel variants are assumed here to arrive bi-annually after Omicron, in which each novel variant is considered 10% more infectious, equally severe, and 10% immune-evading to immunity against infections and diseases compared to the previous one. Original vaccination and subsequent booster coverages are assumed 90% for those at high risk, 80% for those at moderate risk, and 70% for those at low risk of developing severe COVID-19 symptoms. For the baseline ‘no treatment’ scenario, the Supplementary Information presents the additional epidemiological outcomes in Figure S8, the dominant variants in Figure S9, and the underlying age patterns in Figure S10. Additional epidemiological outcomes for all seven scenarios is presented in Figure S11.

The total number of hospitalisations decreases with the introduction of antiviral treatment to varying extents depending on both the treatment efficacy and target population coverage. Treating those at high and moderate risk of developing severe COVID-19 symptoms relative to only treating those at high risk (the current treatment strategy), could reduce annual cumulative hospitalisations by 38% with an antiviral treatment efficacy of 90% and by 14% for an efficacy of 30%. Further expansion of treatment target population by also including those at low risk of severe disease could lead to an additional reduction in hospitalisations of 4% (90% treatment efficacy) and 3% (30% efficacy) (Fig. 1, additional epidemiological outcomes and underlying variant and age patterns in Figures S8–S11 in the Supplementary Information).

**Fig. 2:**
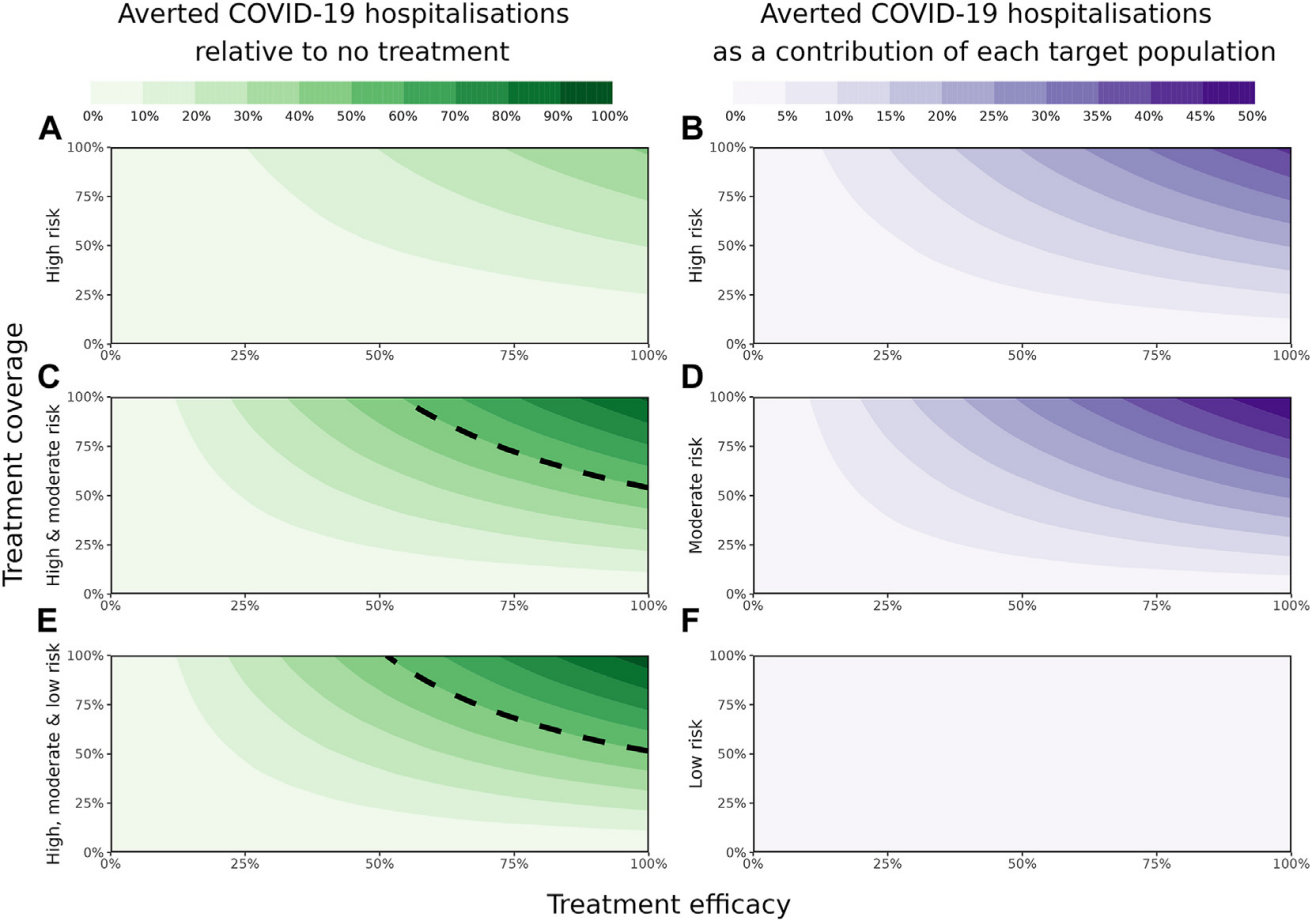
**Percentage of averted COVID-19 related hospitalisations per target population for a full range of coverages (y-axis, 0–100%) and a full range of antiviral treatment efficacies (x-axis, 0–100%), per target population (A, C, E) and as a contribution of each target population (B, D, F)**. The dashed black contour curve represents a 50% reduction in hospitalisations. Figure S12, presents the total number of hospitalisations per 100,000 per year for the three target populations for a full range of treatment efficacy and treatment coverage.

Simulating the full range of antiviral treatment efficacies (0–100%) identified that the total number of hospitalisations generally decreases when treatment efficacy increases (Fig. 2 panels A, C, E, x-axis). The extent of this impact, however, depends on the target population covered. Ranging the treatment coverage (0–100%) amongst the high-risk, high- and moderate-risk, and high-, moderate- and low-risk populations (Fig. 2, panels A, C, E, y-axis), identified that a 50% reduction in hospitalisations (black contour lines in Fig. 2, panels C and E), could not be achieved with treating only the high-risk population, regardless of the percentage coverage and treatment efficacy. However, with 100% coverage of the high and moderate risk population (Fig. 2, panel C), a minimum treatment efficacy of ∼55% could halve the number of hospitalisations. To achieve the same with a 100% effective treatment, covering ∼50% of the target population would suffice. The minimum required antiviral treatment efficacy to halve the number of hospitalisations increases as the treatment coverage reduces. Total hospital and ICU admissions are influenced more heavily by antiviral treatment efficacy rather than treatment coverage (Figure S12).

The biggest contribution to the reduction in hospitalisations can be achieved by treating those at moderate risk relative to treating those at high risk only (Fig. 2, panel D). This is explained by the fact that in a no-treatment scenario, for our assumed demographic setting, the moderate risk population (40–64-year olds) represents the largest population amongst those in hospital (Figure S10). Adding those at low risk of developing severe disease reduces hospitalisations further, but only with a maximum of 5% regardless of the treatment efficacy and total treatment coverage (Fig. 2, panel F).

### Health economic outcomes

**Fig. 3:**
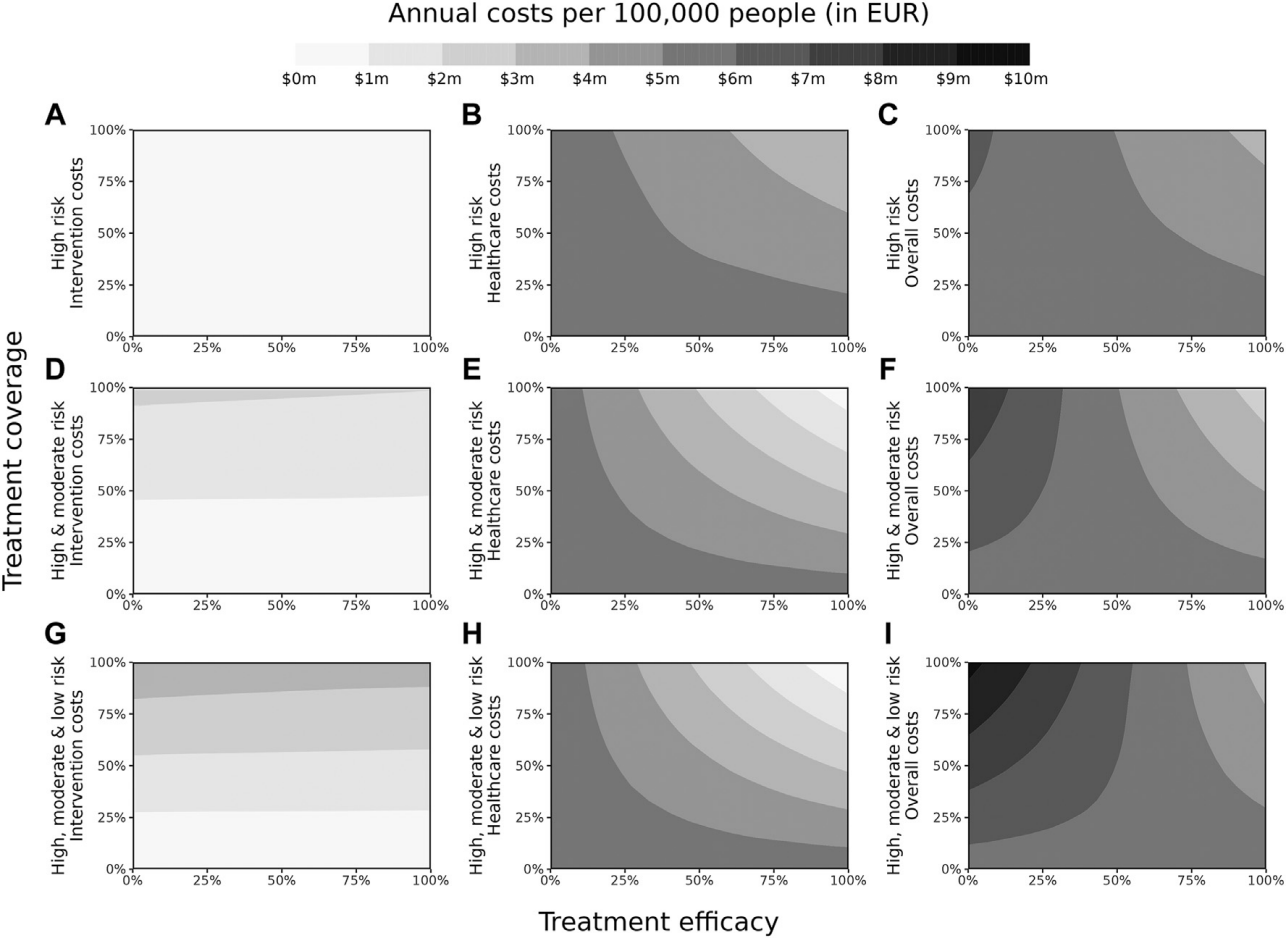
**Representative intervention, healthcare, and total costs per 100,000 people per year for a full range of antiviral treatment efficacies (0–100%), treatment coverages (0–100%) for three different treatment populations; high risk (top row), high and moderate risk (middle row), and high, moderate, and low risk (bottom row)**. Panels A, D, G present the total intervention costs of the antiviral treatment per scenario per 100,000 people per year. Panels B, E, H present the total healthcare costs of hospitalisations and ICU uptakes per scenario per 100,000 people per year. Treatment costs are considered 5% of that of one hospitalisation. Panels C, F, I present the overall costs, adding the total intervention and total healthcare costs together per simulated scenario. Figure S13 presents a global sensitivity analysis for the intervention, health care, and overall costs for the following parameters: treatment coverage, treatment efficacy, treatment cost, primary vaccine coverage, vaccine booster coverage, variant immune-evading capacity, and variant severity.

Intervention costs are completely driven by the antiviral treatment coverage where a higher population coverage leads to higher costs, irrespective of treatment efficacy (Fig. 3, panels A, D, G). The more people take the treatment, the more the total treatment costs. Healthcare costs, however, are primarily influenced by the antiviral treatment efficacy, with healthcare needs and thus costs decreasing as treatment efficacy increases as the antiviral treatment reduces the need for hospitalisation. Additionally, increasing the treatment coverage also reduces healthcare costs, with the effect getting bigger with increased treatment efficacy (Fig. 3, panels B, E, H). Because when more people receive antiviral treatment, less people will need hospitalisation, which leads to reduced healthcare costs. The cumulative costs of both intervention and healthcare costs present an interesting dynamic. The overall costs *increase* when increasing the coverage with low treatment efficacies. This is explained by a higher treatment coverage leading to a need for more treatments, thus higher total treatment costs. However, when these high treatment coverages and costs are combined with low treatment efficacies, they will not reduce the healthcare admissions and thus healthcare costs by the same amount, leading to higher overall costs spent on the increased coverage. On the contrary, the overall costs *decrease* when increasing the coverage with high treatment efficacies (Fig. 3, panels C, F, I). In which case the hospitalisations and associated costs reduce by so much as to compensate for the increased treatment coverage costs.

**Fig. 4:**
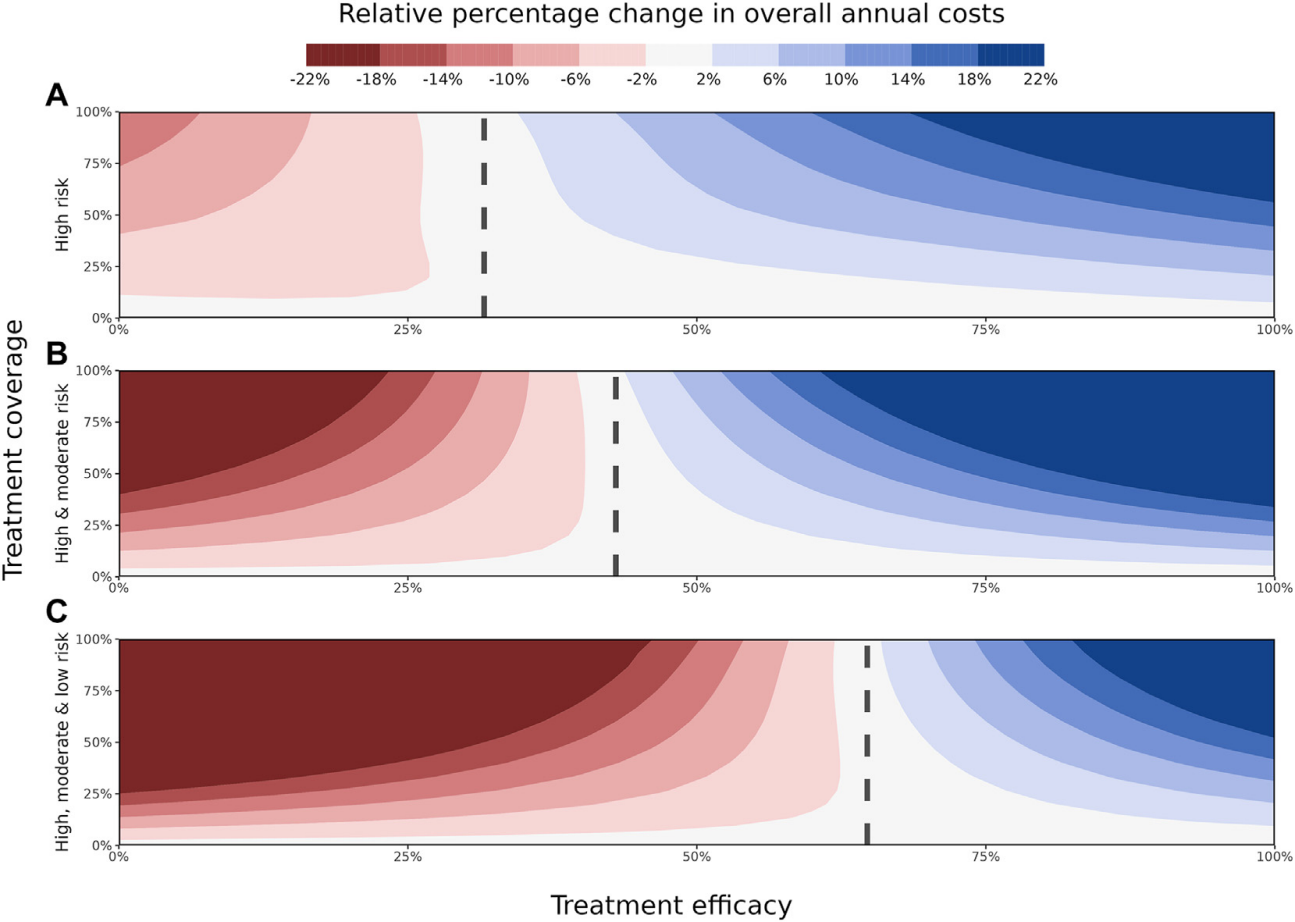
**Heatmap of the relative percentage change in overall costs (intervention costs plus healthcare costs) for a range of treatment efficacies (0–100%) and extended treatment coverages for three different target groups (0–100%), relative to the scenario of no treatment (0% coverage)**. The three different target groups include (A) those at high risk only, (B) those at high and moderate risk, and (C) those at high, moderate, and low risk of developing severe COVID-19 disease. The treatment efficacy “flipping point” is indicated by the vertical black dashed line, at which the strategies go from being more costly (red shades) to cost-saving (blue shades).

The antiviral treatment efficacy threshold for which each strategy becomes cost-saving was found to be ∼30% treatment efficacy for targeting high-risk only, ∼45% for targeting high- and moderate-risk, and ∼65% for targeting high-, moderate- and low-risk groups (Fig. 4). When above this efficacy threshold, the cost savings increase further when treatment coverage is expanded. On the contrary, with antiviral treatment efficacy below these respective thresholds the strategies become costlier when increasing the coverage. This concept provides a treatment efficacy threshold at which the strategies become cost-saving. These strategy-specific efficacy thresholds are agnostic to treatment coverage, where coverage purely determines the extent to which the strategy is costly or cost-saving (Fig. 4, dashed lines).

### Sensitivity analyses

**Fig. 5:**
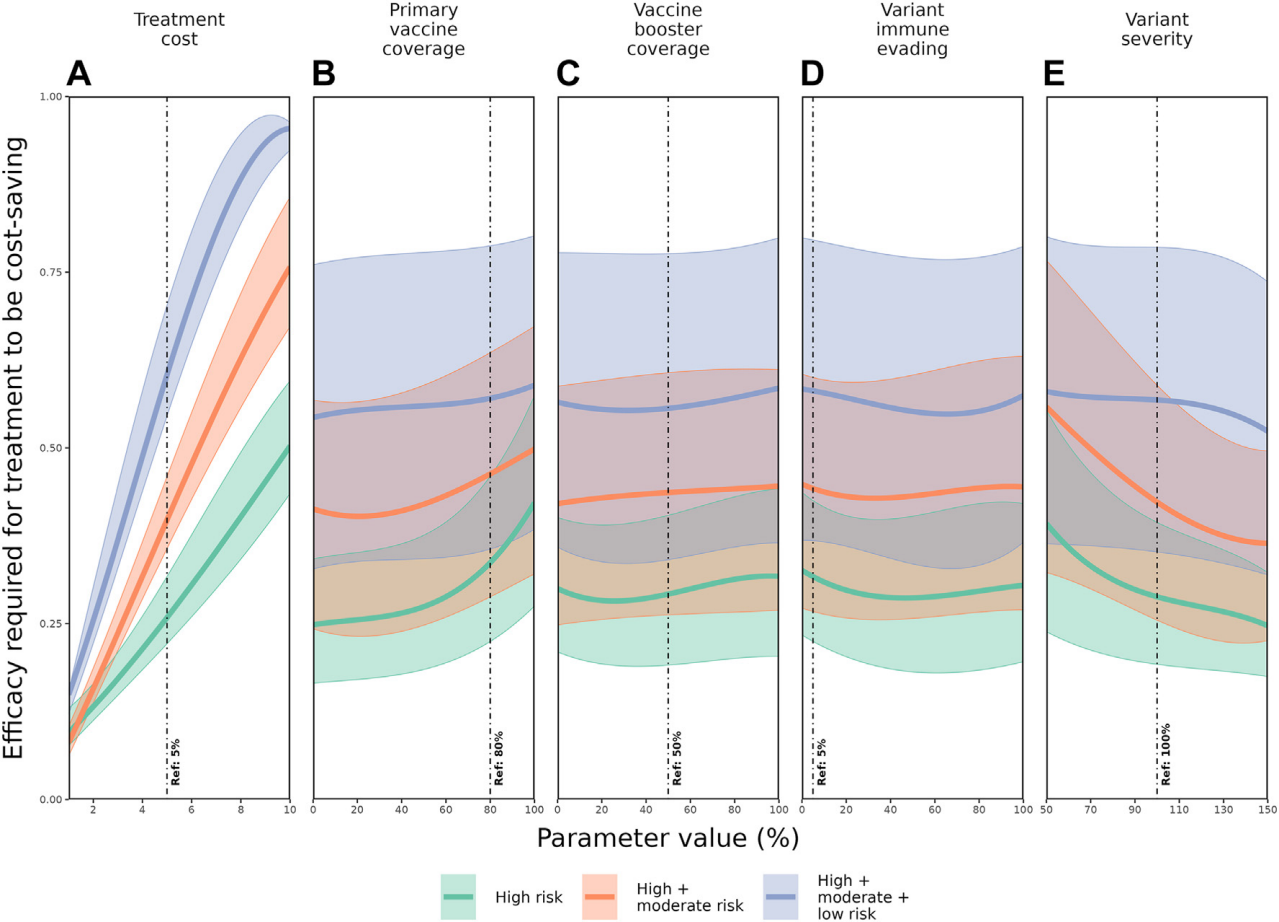
**Global sensitivity analysis of the underlaying influence of the treatment cost(A), primary vaccine coverage(B), vaccine booster coverage(C), variant immune-evading capacity(D), and variant severity(E)on the antiviral treatment efficacy required for a treatment to be cost-effective for three different target populations, represented by the three coloured lines per panel**. The reference values (dashed vertical lines) and their ranges are also presented in Table 1. The shaded regions present the range of model outcomes for each simulated parameter value whilst all other parameters are being varied across their plausible ranges.

The efficacy required for a treatment strategy to be cost-saving (dashed line in Fig. 4) was unsurprisingly found to be highly sensitive to the cost of an individual antiviral treatment, where for higher treatment costs a substantially higher treatment efficacy is required for a strategy to be cost-saving (Fig. 5, panel A). Compared to the effect of treatment cost, the cost-saving efficacy thresholds are relatively insensitive to all other model parameters considered in the analysis (Fig. 5, panels B, C, D, E). This phenomenon becomes more pronounced as the target population is extended to the low-risk group. However, very high primary vaccination coverage and very low emerging variant severity do require a higher treatment efficacy for a strategy to be cost-saving, particularly when only targeting those at high-risk.

The global sensitivity analysis for hospitalisations and associated heath costs is described in the Supplementary Information, Figure S13. We observed that whilst public health outcomes were found to be highly sensitive to emerging variant properties, the cost-saving efficacy threshold findings (Fig. 5, panels D, E) remained robust as they were not particularly sensitive to such variant properties.

## Discussion

With this study we address urgent questions regarding the role and impact of next generation SARS-CoV-2 pharmaceutical interventions on COVID-19, as raised by governments and policy makers.^29^ Within our transmission modelling research framework, we assessed the potential impact of antiviral COVID-19 treatment strategies on public health and health economic outcomes for an extensive range of hypothetical future scenarios. We analysed to what extent the impact is influenced by vaccination coverage, emerging variant properties, and treatment properties. We find that the expansion of treatment coverage amongst the population can substantially reduce hospital costs, which in certain scenarios will counterbalance the increasing intervention coverage costs. The efficacy required for a treatment strategy to be cost-saving (to save more money in averted hospital stays than the cost of rolling out treatment) primarily depends on the cost of the antiviral treatment and the target population's risk of severe disease. Importantly, we find that cost-saving efficacy thresholds are relatively low, and relatively insensitive to epidemiological characteristics such as vaccine coverage and emerging variant properties.

With the treatment scenarios and sensitivity analyses we provide comprehensive quantitative guidance on the potential role and use cases of new antiviral treatments as we enter the endemic phase of SARS-CoV-2. We deliberately did not simulate treatments to replace vaccines, as vaccines that maintain efficacy against current and new variants offer the cornerstone of protection against severe COVID-19.^22^ Therefore, we present the potential of treatment in addition to vaccines, highlighting several scenarios where treatments support reducing COVID-19 hospitalisations and costs in populations with high vaccination coverage rates.

We demonstrate that, in addition to those at high risk of developing severe symptoms (>65 years of age and/or living with co-morbidities), there is also a need to consider access to treatment for those at moderate risk (40–64 years of age), who make up approximately 50% of all COVID-19 related hospital admissions under our demographic and vaccination assumptions and in a scenario without treatment. By expanding the treatment target population to those at moderate risk, hospitalisations can be further reduced substantially. This strategy of targeting the high- and moderate risk group will be cost-saving for treatments with an efficacy of ∼40% and higher, however this is highly sensitive to the cost of treatment. Further extending treatment to those at low-risk of developing severe symptoms (18–40 years of age), led to negligible additional reductions in hospitalisations, while intervention costs increased to such an extent that only very high treatment efficacies combined with low treatment costs could make such a strategy cost saving.

For different scenarios we identified the treatment efficacy at which intervention costs are balanced out by the associated reduction in health care costs. We found this efficacy threshold for a treatment strategy to be cost saving was most influenced by the cost of treatment. While we use representative costs only to explore this relationship, our results on efficacy requirements for a treatment strategy to be cost saving have an important implication. Namely, equitable treatment costs will be crucial to ensure access to preventative health for an individual. From a population and public health perspective, equitable treatment costs will lead to the best possible public health and health economic outcomes.

Should more severe and immune-escaping variants emerge, increased hospitalisations and associated costs are to be expected, emphasising an important use case for antiviral treatments. A major finding of this study is that whilst healthcare and intervention costs are highly sensitive to emerging variant properties, the efficacy threshold for a treatment strategy to be cost-saving is remarkably stable.

While we assessed the potential public health impact of treatments via *immediate* outcomes (averted hospitalisations and ICU uptakes), there will likely be additional *long-term* health benefits because of reduced vascular damage in treated individuals and thus reduced risk of future long-COVID and other cardiovascular outcomes.^30^ A global meta-analysis estimated pooled prevalence of a post COVID-19 condition to be 0.43 (95% CI: 0.39, 0.46), which, besides the additional health burden also leads to increased costs, as these individuals may need further hospitalisations and can be out of the workforce for months.^31^^,^^32^ As more reliable QALY estimates are defined for long-COVID, future modelling studies with longer-time projections could identify the associated reduced health and economic burden attributable to the effects of antiviral treatments on long-COVID. While we demonstrated effective treatments to reduce hospitalisations at individual and public health level, they also reduce the burden on the healthcare system. The demand for COVID-19 care has in the past overshadowed many other health needs, resulting in numerous indirect COVID-19 deaths.^33^ The heightened demand for COVID-19 care has also resulted in the long-term burnout of health professionals and other front-line workers.^34^ While some impacts are not as blatant as averted hospitalisations and deaths, they remain important considerations in the overall effect of COVID-19. Considering these long-term health aversions and impact on healthcare systems, antiviral treatments likely lead to bigger public health outcomes than those presented here.

In contrast to the additional benefits above, previous treatments have also shown potential for less favourable outcomes, where symptomatic rebounds occurred after the treatment regimen finished and individuals had to repeat isolation and treatment procedures, increasing overall costs.^35^ These have not been incorporated in our model. Additionally, drug resistance was also not considered, which could lead to less favourable public health and health economic outcomes than those presented. We did not consider any restrictions around treatment availability, nevertheless, this may be a factor in certain settings, in which case those with the highest risk of developing severe symptoms would need to be prioritised.^36^

Ethical considerations are imperative while interpreting simulations regarding the exposure of individuals to treatment regimens. In reality, some of the very low-efficacy treatments would be unethical to provide to individuals, however, we deliberately simulated the full range of treatment efficacies to provide insight into the trends, rather than simulate reality.

As highlighted above, access to treatment varies geographically and large global inequities are to be expected.^37^ Since the costs of these treatments have generally been set high, which higher-income countries can afford, they serve as a precursor to health inequity. Although treatments have been made widely available, also to low- and middle-income countries, they may not be financially accessible to all, especially not to those who need them most (and are at the highest health disadvantage).^38^

Furthermore, because antiviral treatments are to be taken soon after the onset of symptoms, testing is a prerequisite for use. But COVID-19 tests can be scarce in low-income countries, with more than 3 billion tests performed worldwide, only 0.4% were done in low-income countries, according to the FIND SARS-Cov-2 test tracker. A global roll-out of an antiviral treatment would therefore require a simultaneous massive increase in access to diagnostics.^37^

Our simulations are constructed as an archetypal transmission setting that is intended to be broadly representative of a Northern Hemisphere setting. As such, the results presented in this paper should be interpreted as an overarching understanding of the dynamics that influence the impact of antiviral treatments at population level. In other parts of the world, that differ regarding seasonal patterns, past infection rates, and healthcare systems, the outcomes could prove different. However, the ranging health care parameters presented in this study, such as vaccination coverage rates, and the overlap in past infection rates between settings across the world, make our outcomes still relevant to other parts of the world, when considering drivers of antiviral treatment cost-effectiveness. The results aim to aid discussions around both the development as well as use cases of antiviral treatments in the future of COVID-19, rather than show future scenarios for specific countries and settings.

With our findings we provide quantitative guidance for antiviral treatment use cases for a range of hypothetical future scenarios. To highlight, antiviral treatments can substantially reduce hospitalisations even in high vaccination coverage scenarios and at times of lower incidence. High levels of future variant immune-evasion increase the need for antiviral treatments to reduce hospitalisations. In addition to treating those at high risk of developing severe COVID-19 we show that also treating those at moderate risk could avert substantial hospitalisations. This strategy is cost-saving (for a treatment cost of €500) from a treatment efficacy of around 40%. These findings are robust against a wide range of epidemiological variables. For a treatment to be cost-saving, the biggest influence is treatment cost, and therefore equitable treatment costs are crucial in achieving the best possible public health and health economic outcomes at population level across the full range of health systems.

## Contributors

EALR, AJS and MAP conceived and conceptualised the study design. EALR, IM, and SG performed literature searches. AJS developed and updated the model. AJS, EALR, and IM accessed and verified the underlying data. EALR, AJS, and IM performed the formal analyses, prepared figures, and conducted model and analysis validation. EALR wrote the draft manuscript. SG reviewed and edited the manuscript. All authors contributed to interpretation of the results, writing of final version of the manuscript, and gave final approval for publication.

## Data sharing statement

All model code and Figure code are open access and publicly available at https://github.com/SwissTPH/OpenCOVID/tree/manuscript_treatment. Model and figure code for this study use version 4.2 of OpenCOVID.

## Declaration of interests

Payments made by World Health Organization Immunization, Vaccines and Biologicals department to Andrew Shattock as an independent consultant working on a project focused on vaccine impact for a portfolio of diseases that did not include COVID-19. The other authors declare no competing interests.
