# Supplementary material for: Efficacy thresholds and target populations for antiviral COVID-19 treatments to save lives and costs: a modelling study
Source: eClinicalMedicine. 2024 Jun 21;73:102683. doi: 10.1016/j.eclinm.2024.102683 (PMC11246010; doi:10.1016/j.eclinm.2024.102683)
Supplement: Supplementary Information [file mmc1.docx]

**Supplementary Information**

The following descriptions, figures, and tables supplement the manuscript:

**“Efficacy thresholds and target populations for antiviral
COVID-19 treatments to save lives and costs”**

Epke A Le Rutte, Andrew J Shattock, Inês Marcelino, Sophie Goldenberg, and Melissa A Penny

**Supplementary Information**

**Contents**

[1. Supplementary methods 3](#_Toc157507974)

[1.1. OpenCOVID 3](#_Toc157507975)

[1.2. Additional model descriptions 5](#_Toc157507976)

[3.1. Intervention parameters 11](#_Toc157507977)

[3.2. Sensitivity analysis - supplementary details 13](#_Toc157507978)

[4. Supplementary Figures 14](#_Toc157507979)

[5. Supplementary Table 21](#_Toc157507980)

[6. Supplementary discussion 22](#_Toc157507981)

[7. References 24](#_Toc157507982)

# Supplementary methods

## OpenCOVID

*History*

OpenCOVID is a stochastic, discrete-time, individual-based simulation model of SARS-CoV-2 transmission and COVID-19 disease (**Figure S1**). The model was originally a deterministic compartmental model developed in early 2020 for the European Centre for Disease Prevention and Control in response to the COVID-19 outbreak in Europe. In this early phase, the model was simultaneously calibrated to all EU member states, and was used to support several EU risk assessments and policy briefs. In late 2020, the model was converted from a population-based to an individual-based structure, and repurposed to specifically represent Switzerland^1^. Over the course of 2021, the model was used extensively to provide policy support for the Swiss Federal Council via the Swiss National COVID-19 Science Task Force. In 2022, the model calibration process was extended to pseudo-represent archetypal, generic settings to address setting-agnostic questions. In previous publications OpenCOVID has been applied to identify; 1) the impact of vaccination and non-pharmaceutical interventions on SARS-CoV-2 dynamics in Switzerland ^1^, 2) the impact of future emerging variants for a wide range of scenarios^2^, and 3) the frequency, timing, and target groups for future COVID-19 vaccination booster strategies ^3^.

*Brief model description*

The model has been described in detail in Shattock, Le Rutte *et al*., 2022^1^. In brief, once an individual becomes infected within the OpenCOVID framework, the person is assigned with the viral variant of the infector, after which the individual enters a latent phase, during which the person is infected but not yet infectious. The infected individual then becomes infectious and remains either asymptomatic or will develop mild or severe disease. Individuals that develop severe disease may, after some time, either seek hospital care or remain outside the hospital setting (e.g., within care homes). Three distinct prognosis tracks are modelled for those that seek hospital care: 1) the patient will eventually recover without intensive care, 2) the patient will require intensive care but will eventually recover, and 3) the patient will require intensive care and will ultimately die from COVID-19-related complications. See **Figure S1** for an illustration of the modelled natural history and prognosis pathways. After infection and recovery, the person will enter a state of immunity, which wanes over time (immunity patterns are described in more detail in section 1.2).


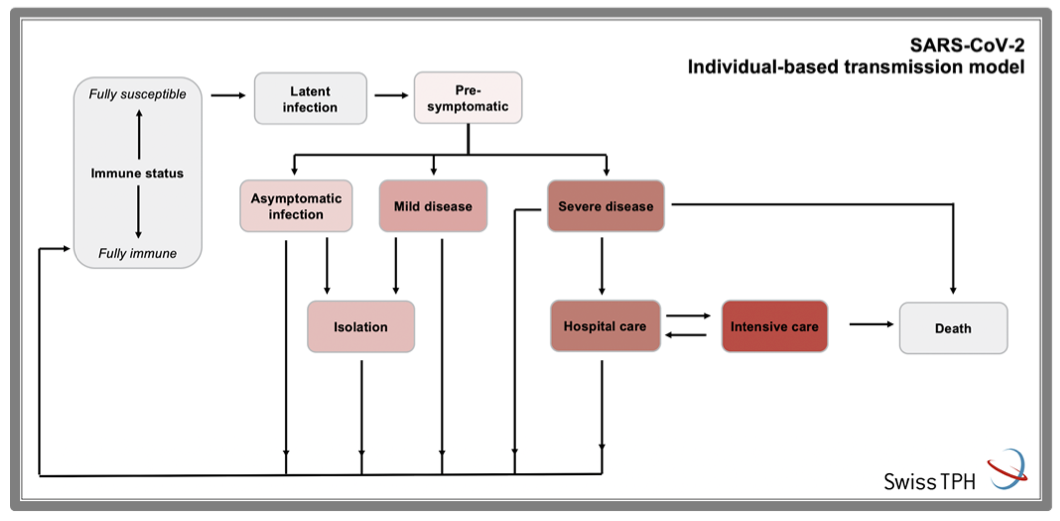


**Figure S1. Schematic of OpenCOVID model structure.** The model captures potential states of individuals.
The ‘immune status’ ranges from fully susceptible to fully immune, where any level of immunity is a consequence of previously acquired natural immunity and/or vaccination. Other states include latent infection, pre-symptomatic, and the asymptomatic state from which vaccination may also lead to development of immunity. After infection, some remain asymptomatic, while for others either mild or severe disease progression
may occur. Isolation or care (hospital care, intensive care) may be required for those with symptomatic infection, resulting in recovery or death. Increasingly darker shading (grey, pink, red, dark grey) indicates increasing severity.

*Open access code for the model and simulations*

Open access source code for the OpenCOVID model is publicly available from the OpenCOVID git repository, github.com/SwissTPH/OpenCOVID/tree/manuscript_treatment. ^4^ The code has been licensed under GNU General Public License v2.0 and is freely available for use or modification, including further development or independent development. OpenCOVID is written primarily in the R programming language and is stable with R version 4.1.0. The source code requires cluster access to perform several computationally expensive tasks; as standard the model is configured to run on sciCORE, the scientific computing cluster at the University of Basel. In terms of running the source code, an important note is that ’OpenCOVID’ is an umbrella term for 1) a transmission model, and 2) a full A → Z pipeline that allows the user to calibrate the model to some setting, simulate a series of scenarios, and produce a set of standard results. The general concept is that the user can simulate the model, assess the outcomes, and draw conclusions without the need to write any R code at all. For more information about how to run the source code, see the readme file in the OpenCOVID git repository. ^4^

## Additional model descriptions

*Model calibration and validation*

In the calibration and validation process the model is fitted to user-defined input data such as temporal epidemiological data or an initial effective reproductive number, denoted Re (which was used in this study). The aim of the calibration process is to determine a ’best fit’ parameter set. The ’best fit’ parameter set is defined as the one which minimizes the value of a predefined loss function.

The first step in the calibration is to initialize the model by sampling across the parameter hyperspace, which is the region defined by the bounds of each calibrated parameter. This is accomplished by using a Latin hypercube sampling algorithm to ensure equal exploration of the entire parameter space, without focusing on regions where parameter priors are located. A total of Npar unique locations are sampled. Each parameter set is then simulated Ninit times for different stochastic realizations using OpenCOVID, and the model outputs are recorded. All model simulations are run in parallel on a high-performance computing cluster. The log-likelihood objective function is then calculated for each parameter set to assess the quality of fit between the model outputs and the epidemiological data.

Following the initialization step, Npar unique locations are randomly split into a training set and a test set. A Gaussian Process model is then trained using the training parameter sets and associated likelihood values to learn the relationship between the parameter set and the log-transformed objective functions given a model parameter set. For this analysis, we used a heteroscedastic Gaussian Process algorithm as model emulator ^5,6^. Model fit is then **validated** using the test set which was set aside earlier in the process. Ten rounds of adaptive sampling were applied to efficiently resample regions of the parameter hyperspace that were good candidates for the global optimum. An expected improvement acquisition function was used to identify these candidate regions and sample 100 new parameter sets per round, with a filtering function applied to ensure resampled parameter sets are not within a predefined distance of each other (with distance measured in Manhattan units). With the newly sampled parameter space, the model emulator is re-trained. Finally, the optimal parameter set that achieves the best quality of fit is determined via an adaptive stochastic descent algorithm that operates on the (re-)trained model emulator.^7^

Fitting to country epidemiological data: OpenCOVID can be calibrated to setting specific - national or subnational - epidemiological data. Model outputs can be matched to six types of observed temporal metrics: 1) daily confirmed COVID-19 cases, 2) daily COVID-19-related deaths, 3) daily hospital admissions, 4) daily ICU admissions, 5) COVID-19 hospital occupancy, and, 6) ICU occupancy.

Fitting to Re derived from data or user-defined, which was used for the purpose of this study: The model can represent any transmission intensity (Re) by identifying the average number of contacts to arrive at a certain Re. Uncertainty in transmission intensity is captured by varying the average number of contacts such that Re is within +/- 10% of the calibrated value. For this study, the model is initialised in spring 2022 with an Re of 0.9, with 65% of the total population considered to have been previously infected, broadly representative of a endemic European setting. ^8^

*Prognosis probabilities*

Once infected, a prognosis is derived for all individuals by stochastically sampling from a uniform distribution. Numerous factors influence the prognosis of a newly infected individual, including their age (**Figure S2**), any co-morbidities, the severity of the viral variant they are infected with, and their immunity to disease through a potential infection history and/or vaccine status (**Figure S3**). Once a prognosis is assigned, the associated outcome (death or recovery following asymptomatic, mild, severe, or critical disease) can be altered only by effective antiviral treatment.^1–3^

*
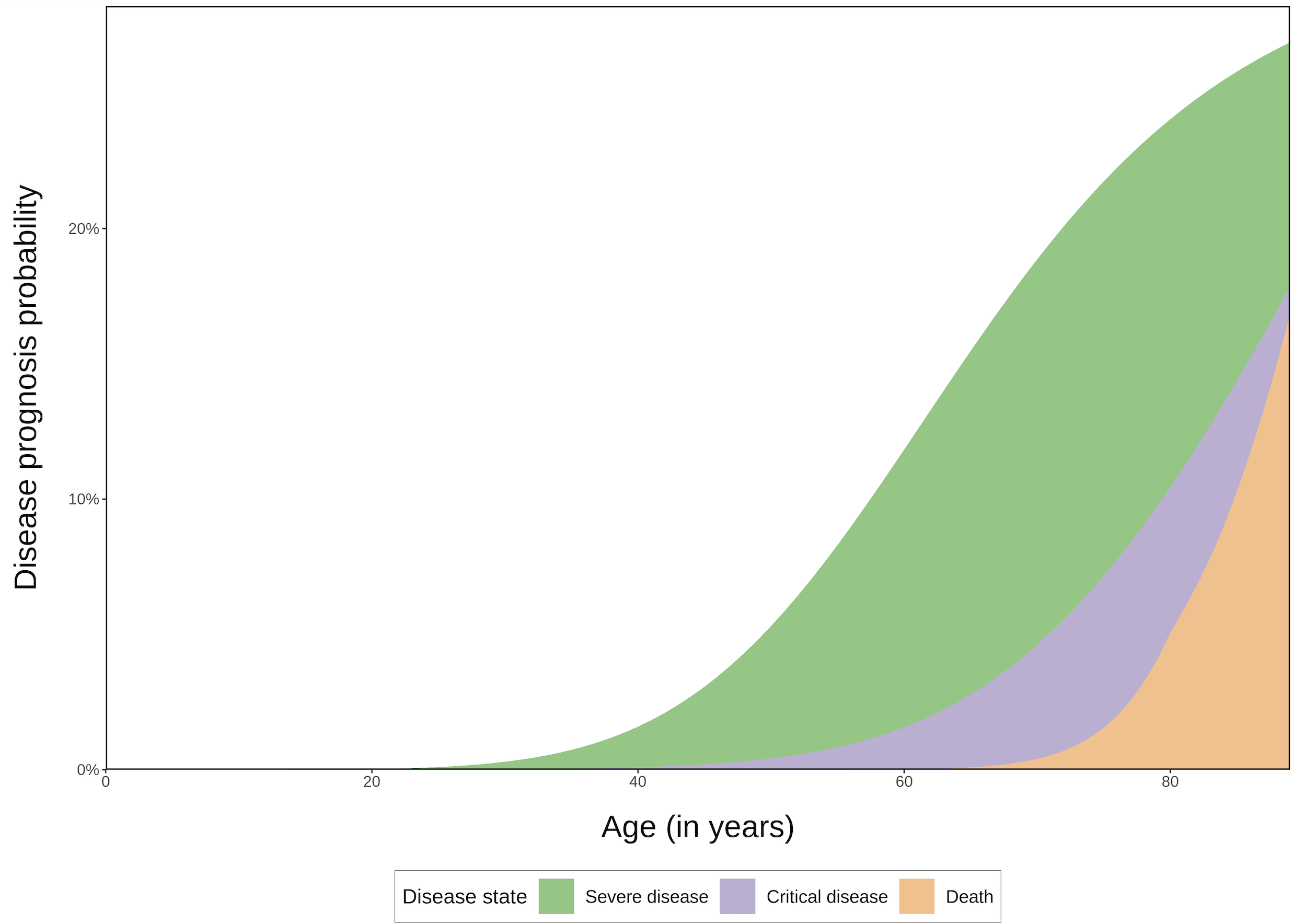
*

**Figure S2. Default distributions of disease prognosis probability by age in OpenCOVID.**

**
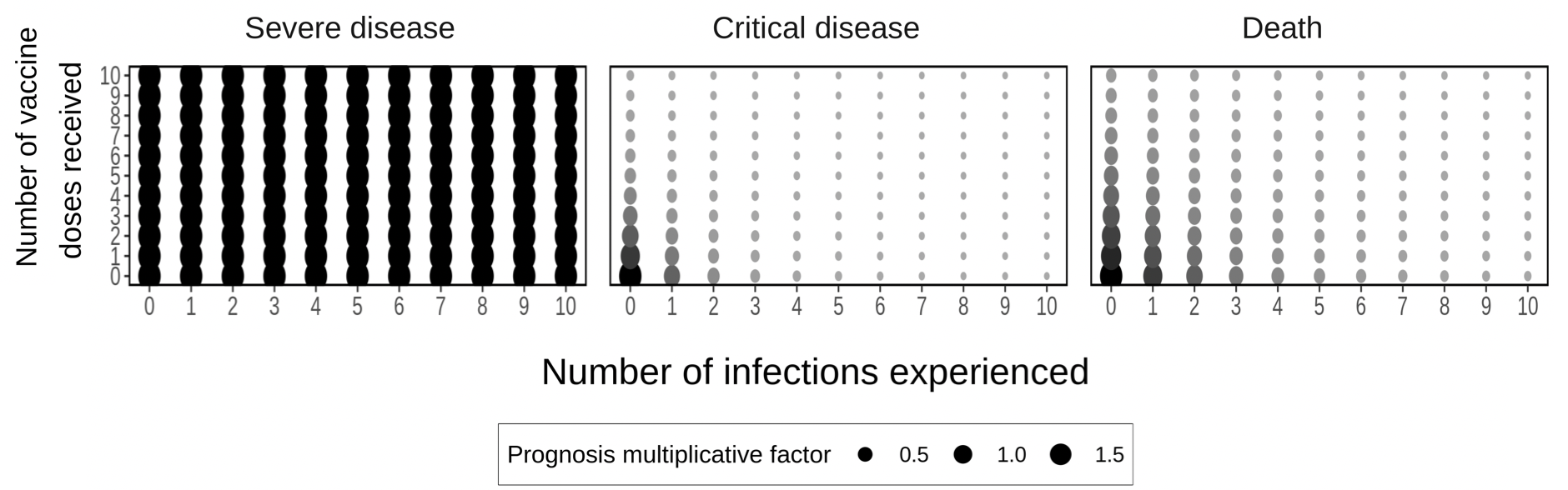
**

**Figure S3. Y-axis: Number of COVID-19 vaccine doses received (0 to 10), X-axis: number of infections experienced (0 to 10) by disease state (severe, critical) or death for a newly emerged (novel) SARS-CoV-2 variant by prognosis multiplicative factor.**

*Viral load*

The viral load of each infected individual is time-varying and depends on the time since infection. We directly associate the viral load at a given time with infectiousness of that individual. Following an initial latent period after infection for which we assume viral load to be zero (and therefore that the infected person is not yet infectious), we represent viral load using a gamma probability density function for the remainder of the infectious period (**Figure S4**). As we assume infectiousness to be proportional to viral load^9^ we standardise the viral load values to be between zero and one. Thus, we convert the viral load into an infectiousness scaler, which scales the probability that the infected individual can infect their contacts. Figure S4 illustrates this infectiousness scaler profile from the time since infection using a gamma distribution with shape parameter α= 3 and rate parameter β = 0.5, which are the default parameters used in the model. These parameters were selected to best represent the current understanding of viral load profiles from time since infection^10^.

*
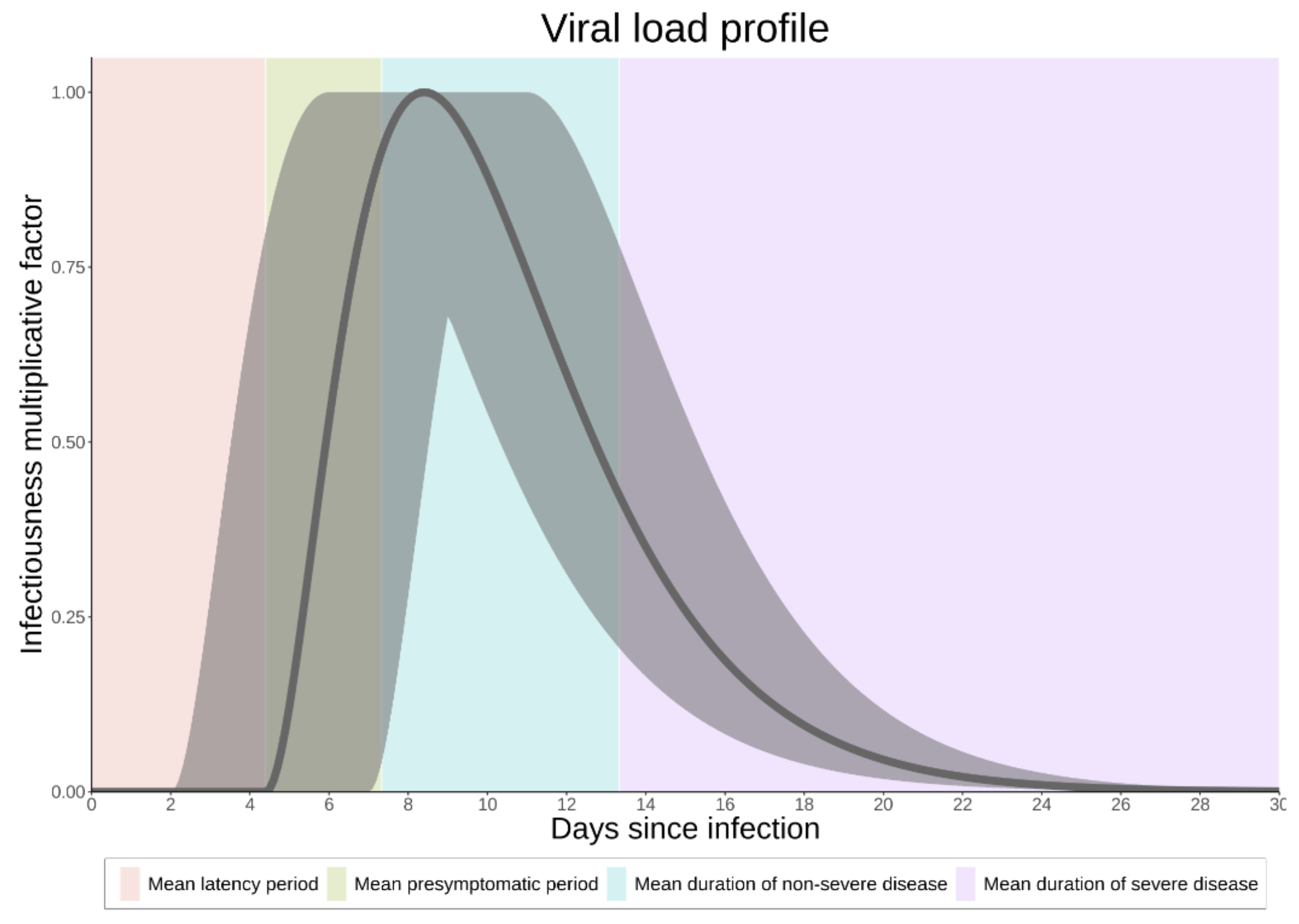
*

**Figure S4: Viral load profile in OpenCOVID. Curve is standardised between zero and one to yield an
infectiousness multiplier used to calculate the probability of transmission.** Peak infectiousness is generally
reached around the time of symptom onset.

*Viral variants*

OpenCOVID is able to represent the introduction and propagation of multiple viral variants into and within the population. The emergence of such variants is not dynamic; that is, variants must be imported into the population at a pre-defined time. Once imported, however, the propagation of such variants is stochastic. ^6^

Novel variants may differ in three viral properties from already circulating strains:

1. Infectivity (multiplier of transmission probability per exposure)
2. Severity (multiplier of probability of developing severe disease per infection)
3. Immune-evading capacity (percentage reduction of pre-existing immunity)

All three properties are defined relative to those of the currently dominant variant.
The infectivity and immune-evading properties can affect the probability of transmission, whilst the severity property affects the probability of developing severe disease. The immune-evading capacity exclusively affects the exposed individual’s immunity to infection (i.e., their susceptibility), thus only impacting the rate of new infections and not influencing severity once infected. The immune escape capacity of a variant may affect any of the two sources of immunity (that is, immunity through infection and vaccination) depending on the infection and vaccination status of the exposed individual.

In this study, new variants are assumed to arrive once yearly ahead of peak winter. In our reference parameterization, such variants are considered to be 5% more infective, equally severe, and 5% immune-evading relative to the previous variant. ^11^ Further variant assumptions are addressed in the sensitivity analysis.

*Seasonality*

In OpenCOVID we represent a reduced probability of transmission per contact during warmer
periods, reflecting a larger proportion of people coming into contact outdoors where the probability
of transmission is lower. Conversely, cooler periods represent an increased transmission probability,
reflecting a larger proportion of contacts being in closer contact indoors where the probability of
transmission is higher. To represent such an annual cycle, we model a seasonality scaler, denoted σ, using a cosine curve with an additional scaling parameter (**Figure S5**). The resulting temporal curve is applied as a multiplicative factor in the transmission equation to reflect the effect of temperature on transmission probability per contact. Figure S5 illustrates the seasonality scaler for three parameterisations of seasonal effect. The grey curve is used for the purpose of this analysis.

**
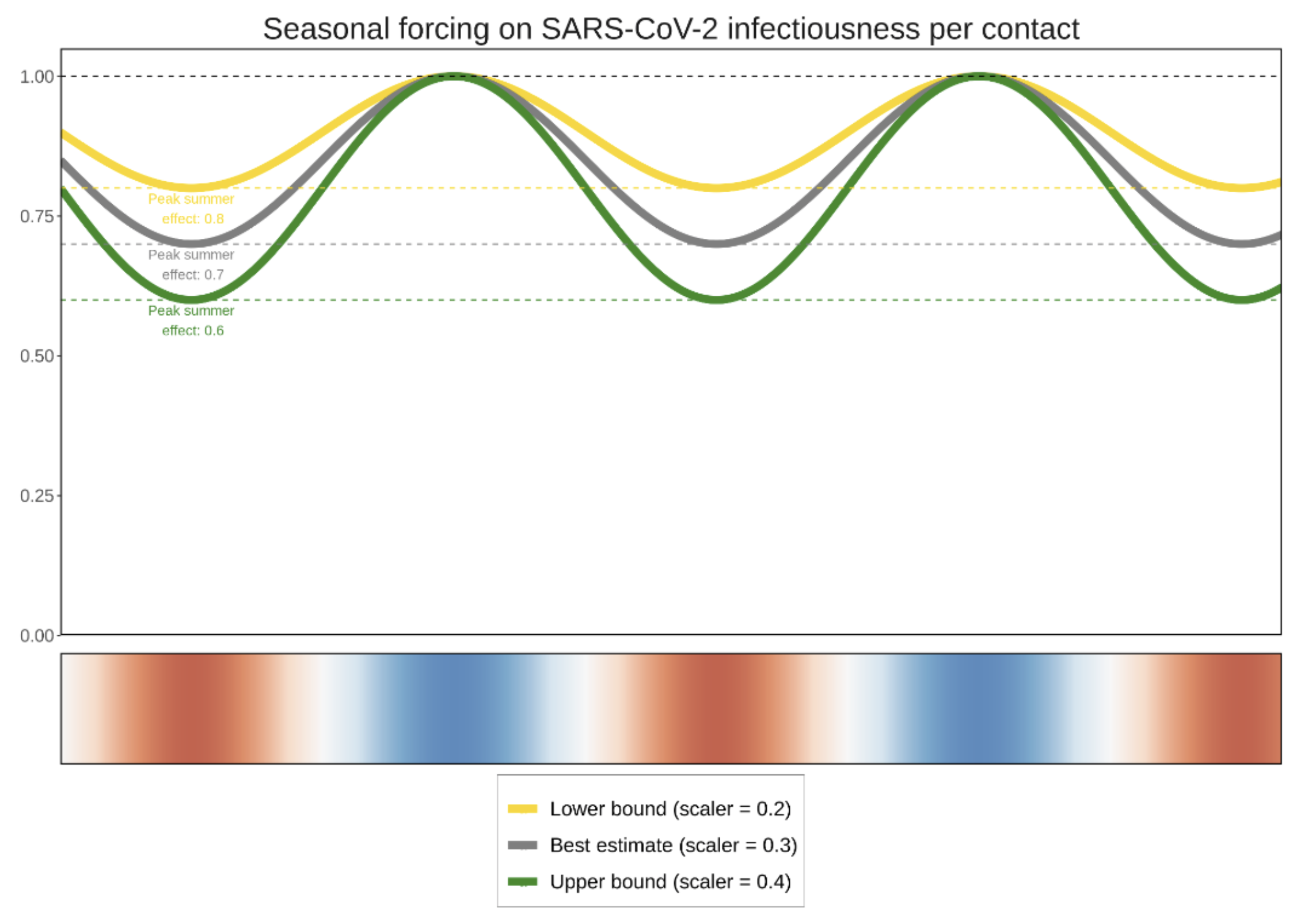
**

**Figure S5: Impact of seasonal forcing scalers (best estimate (grey curve), lower bound (yellow curve), and upper bound (green curve)) on SARS-CoV-2 infectiousness per contact over a two-year period.** Seasonality is illustrated in the bottom row where red shading indicates the warmer spring and summer seasons, blue the cooler fall and winter seasons, and white the seasonal transition periods.

*Immunity*

OpenCOVID represents two mechanisms of immunity. An infection-blocking mechanism and a symptom and severe disease blocking mechanism. Both can be induced through one (or more) of three processes:

1. SARS-CoV-2 infection (naturally acquired immunity)
2. COVID-19 vaccination and consequent booster doses (vaccine induced immunity)
3. Pre-exposure prophylaxis (PrEP) with monoclonal antibodies (mAb induced immunity)

In each case, immunity is considered to wane over time as illustrated in **Figure S6**. In cases where the susceptible individual has partial infection-blocking immunity from multiple sources (also described as hybrid immunity), the maximum value only is taken to represent total immunity to infection. That is, hybrid immunity is represented in the simplest sense. Dependent upon the viral variant with which the susceptible individual is exposed to (that is, the viral variant with which the infectious individual is infected with), immunity (both infection-blocking and disease-blocking) may be reduced by a multiplicative scaling factor.


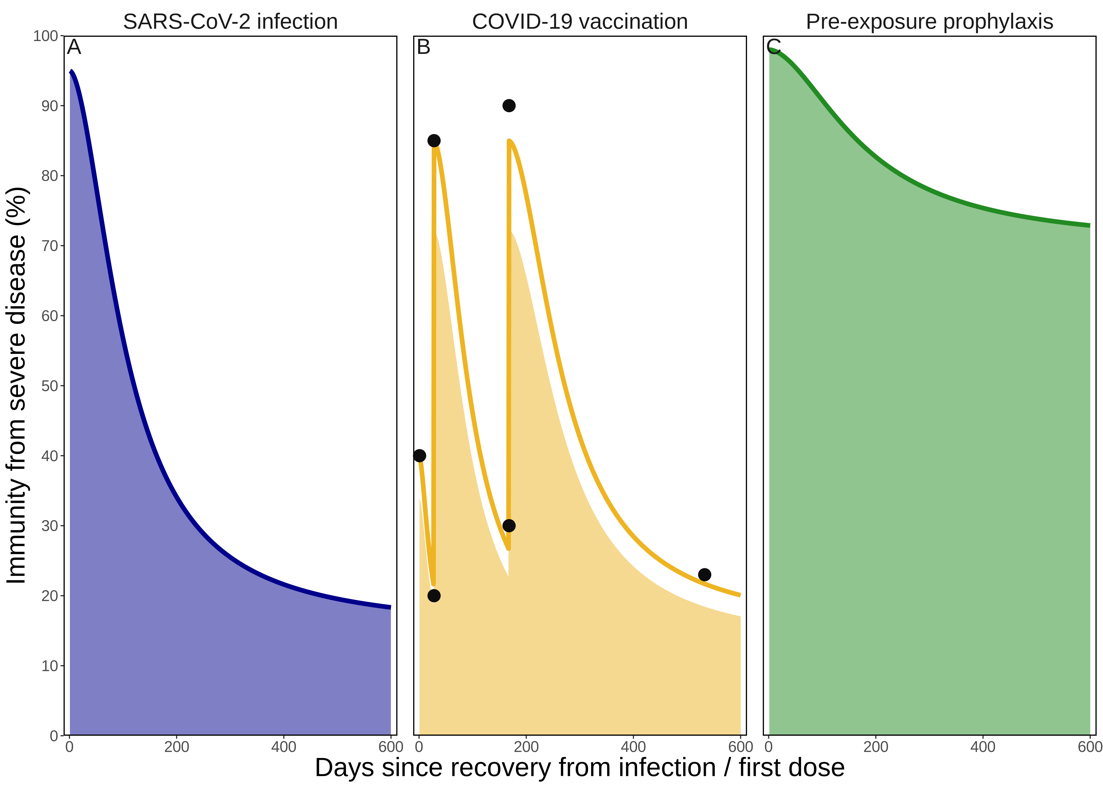


**Figure S6. Profiles of naturally acquired immunity following infection with SARS-CoV-2 (panel A), COVID-19 vaccine induced immunity (panel B), and immunity following pre-exposure prophylaxis (panel C).** Points shown in panel B indicate initial vaccine efficacy following primary vaccination (doses one and two) with exponential decay, with a rebound in efficacy from subsequent booster doses followed by identical exponential decay based on Andrews *et al*., 2022 ^12^.

## Intervention parameters

The model by default simulates the transmission dynamics of SARS-CoV-2 in a population without
any interventions besides the availability of diagnostics and hospital and ICU care. This section
provides an overview of the two interventions that have been introduced into the population in this study. Other interventions that have been implemented in OpenCOVID but that are not used here include; testing, diagnosis and isolation strategies, non-pharmaceutical interventions such as social distancing and facemask use, and pre-exposure prophylaxis with monoclonal antibodies. The impact of the interventions can be analysed at population level and interventions can be compared against each other regarding their impact on public health parameters (e.g. incidence, hospitalisations, deaths) and cost-effectiveness.

*Treatment*

In the model, once an individual receives antiviral treatment, it reduces their probability of developing severe symptoms, of which the extent depends on the treatment efficacy. Adjustable treatment parameters include: treatment efficacy, treatment coverage per risk group, start date of treatment availability, duration of scale-up to maximum capacity, and total treatment dose availability.

The mechanism behind the treatment efficacy (x percentage) means that x of people, out of 100 that take the treatment, have their viral load fully cleared. In 100-x of individuals it will have no impact.
We assume the treatment efficacy to reduce the risk of hospitalisation or death. Individuals are only
eligible for treatment if they have been diagnosed with a SARS-CoV-2 infection.

Everyone who gets infected in the model is assumed to be diagnosed and then eligible for treatment depending on their risk group coverage and treatment availability. The model then also considers a probability of those testing positive for SARS-CoV-2 actually taking the antiviral treatment that is available to them. There is an assumed 2-day delay between the start of symptoms and being diagnosed, followed by a 3-day delay to taking the treatment. This leads to the treatment taking effect 5 days after symptom onset. Those successfully treated return to the recovered stage and are once more
susceptible to infection. We assume that the immunity of a person who has been successfully treated
to be the same as that for a person who naturally recovered. This may not be fully representative
but was modelled accordingly for simplicity. Treatment coverage can be differentiated by priority
group.

*Vaccination*

In OpenCOVID, vaccines have a two-fold effect in the vaccinated individual. First, they provide protection against new infection through development of immunity (infection-blocking effect). Second, once infected, vaccines reduce the probability of developing severe symptoms (disease-blocking effect), leading to reduced hospitalisations,
intensive care unit admissions, and potentially death. Pre-defined sub-populations can have their
own vaccination coverage rate (e.g. to reflect a higher vaccination coverage rate in older people).
Individuals who received initial vaccination (doses one and two) are considered eligible to receive
vaccination booster doses at any selected frequency, booster acceptance probability, and booster
drop-out rate. All parameter values can be specified per sub-population. Vaccine and booster
related immunity and associated waning profiles are described in the immunity sections above (4.4
Immunity to infection and 5.5 Immunity to disease).

For the treatment study, the initial vaccination coverage rates are set at 80% for each
of the high, moderate, and low risk populations.^13^ These are followed by yearly boosters, in
which we assume a probability of booster dose acceptance of 50% relative to the original
vaccination coverage rate. Additionally, a booster drop-out rate is defined, in which we assume a
further 10% of individuals to drop-out of the booster coverage rate across all risk groups, per
booster round. Primary vaccination coverage and booster coverage are both varied in the sensitivity
analysis.

*Healthcare costs*

Healthcare costs are representative only as we later refer to treatment costs relative to hospital costs. Costs were calculated as a single cost per person for one full hospitalisation and/or ICU admission. Hospitalisation costs were based on a health care cost model by Czernichow et al. (2021)^14^. The authors used data of health costs from Denmark, France, Spain, and the UK in publicly funded hospitals with an average hospitalisation cost per day of €883, ICU cost per day of €1925, and €3183 for ICU with intermittent mandatory ventilation. We assumed a normal distribution with a mean of 9 days in hospital and 7 days in ICU, both with a standard deviation of 1 day. Per person we assumed a representative total cost of **€10,000** per hospitalisation and **€20,000** per stay in ICU. ^15^ For these representative costs, the current cost of one treatment regimen (€500) is about 5% of that of a hospital stay. Adding the intervention and healthcare costs together leads to the total costs concerned.

## Sensitivity analysis - supplementary details

We conducted the sensitivity analysis by sampling 1000 parameter sets from the 7-dimensional parameter space using Latin Hypercube sampling. Each parameter set was simulated using the transmission model 10 times to account for stochasticity in model outcomes.

To assess the effect of each parameter on each model outcome of interest in a continuous manner, we trained surrogate model emulators using the sampled parameter sets and associated model simulation outputs. A surrogate model emulator was trained for each model output of interest (intervention costs, healthcare costs, overall costs, and cost-saving efficacy threshold) via a Gaussian Process approach. See **Figure S7** for diagnostic plots of the model emulator, illustrating the predictive ability of each emulator. To assess how sensitive each model outcome is to a given model parameter, we use the corresponding emulator to determine the range of the model outcome for monotonically increasing values of the given parameter whilst also varying all other parameters across their plausible ranges.


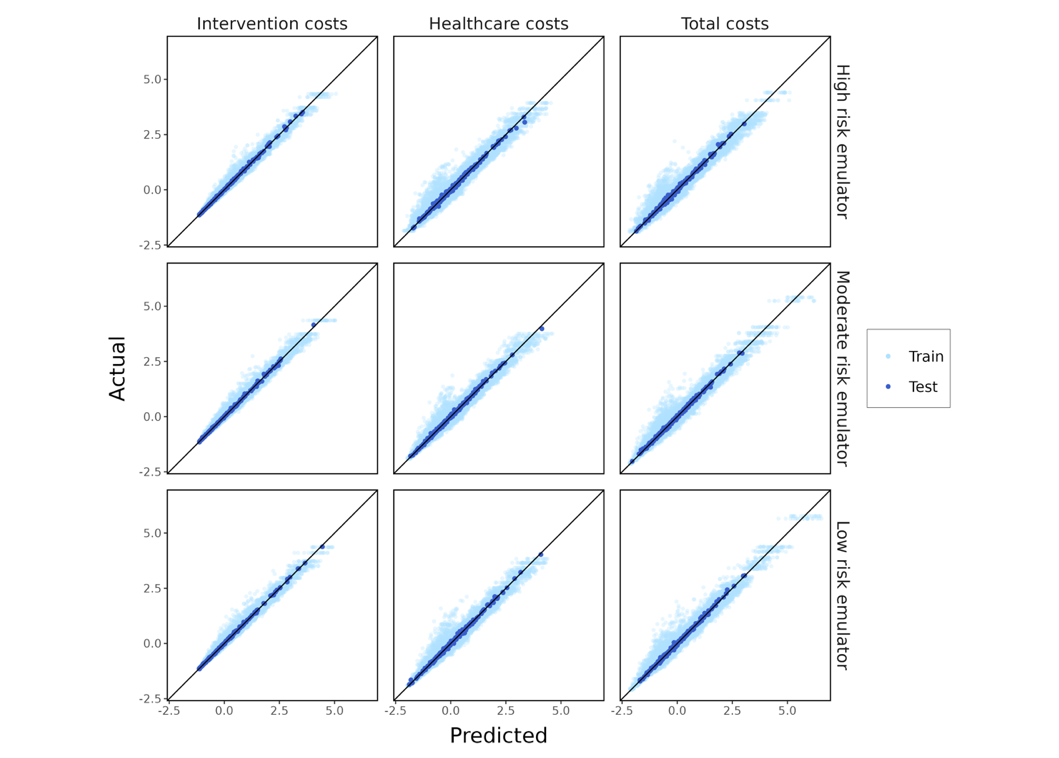

**Figure S7. Model emulator predictions for all three risk groups showing the actual and predicted values**. The model emulator is trained for each model output assessed (hospital admissions, ICU admissions, intervention costs, healthcare costs, overall costs) via a Gaussian Process approach.

# Supplementary Figures


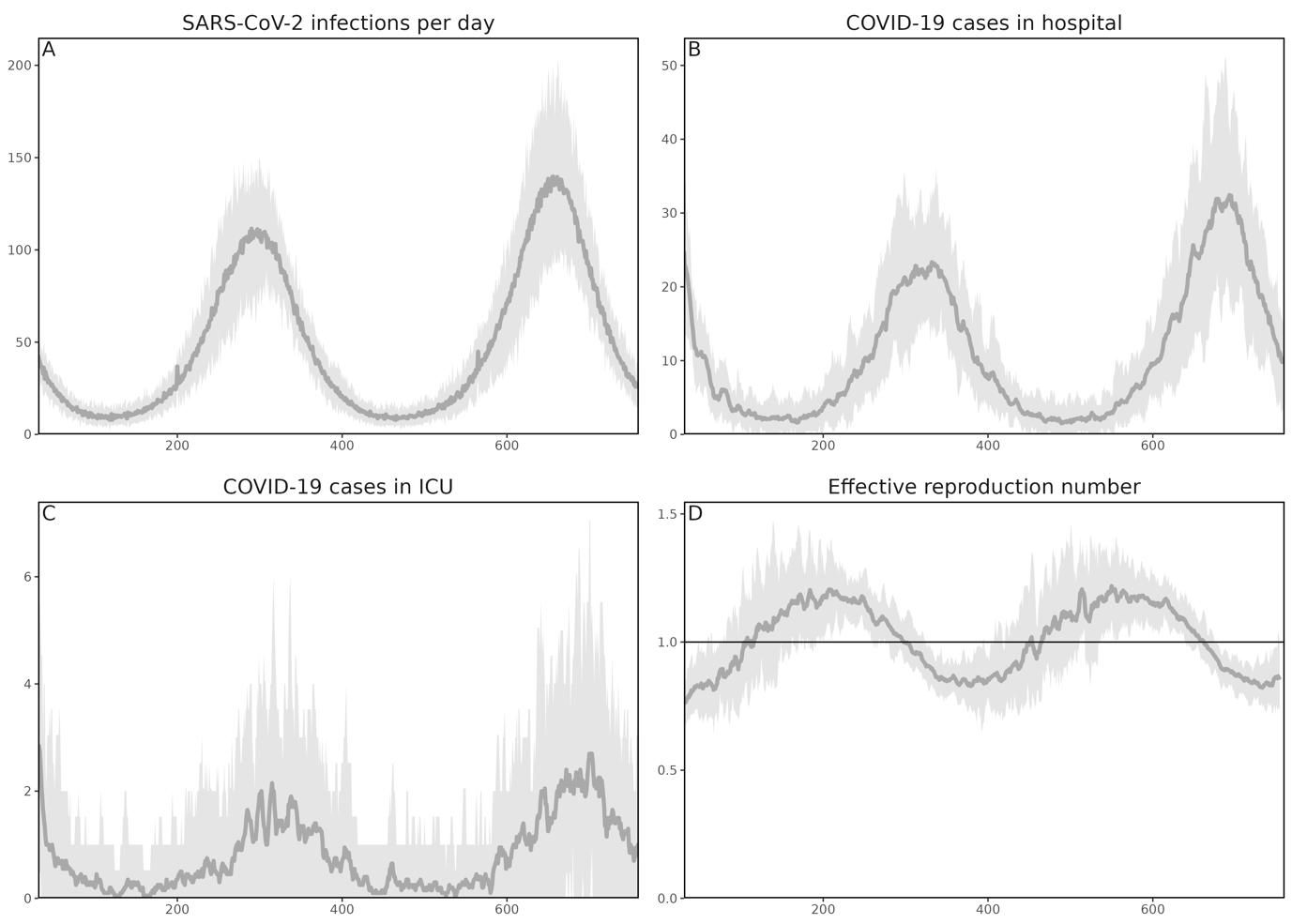


**Figure S8. Two-year temporal pattern for the “no treatment” scenario of Figure 1, showing additional epidemiological SARS-CoV-2 outcomes for a simulated population of 100,000.** The waves reflect the seasonal pattern. In panels A, B, and C, the second wave is slightly higher than the first because of the annual arrival of novel emerging variants as illustrated in **Figure S9**. The uncertainty bounds represent 95% prediction intervals from 50 simulations per modelled scenario, capturing both parameter and stochastic uncertainty.


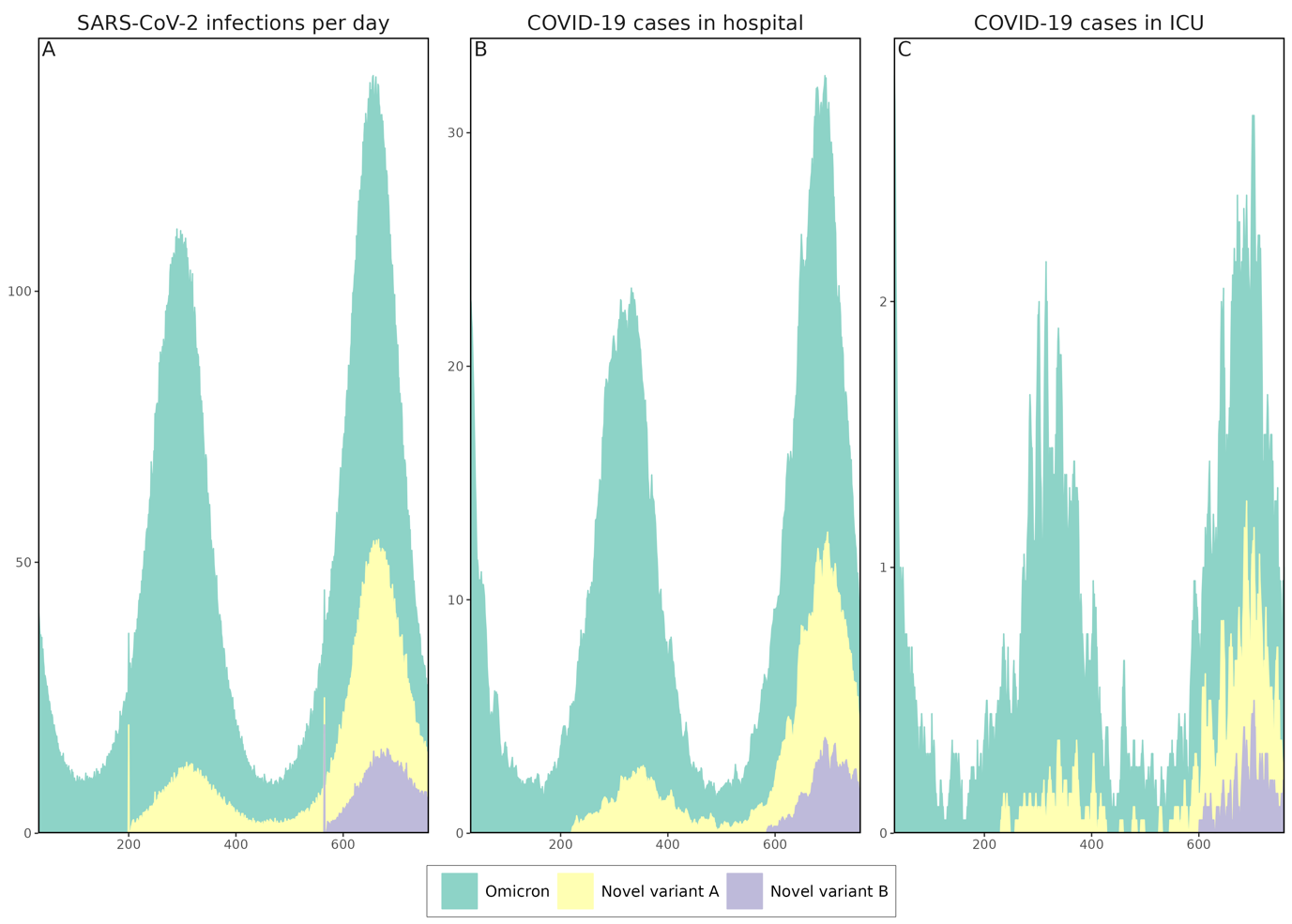


**Figure S9. Two-year temporal pattern for the “no treatment” scenario of Figure 1, showing the underlying variants for A) SARS-CoV-2 infections, B) COVID-19 cases in hospital, and C) COVID-19 cases in ICU for a simulated population of 100,000.** Novel variants are assumed here to arrive annually after Omicron, in which each novel variant is considered equally severe, 5% more infectious, and 5% immune-evading to any immunity against infection and disease acquired against the previous variant. The uncertainty bounds represent 95% prediction intervals from 50 simulations per modelled scenario, capturing both parameter and stochastic uncertainty.


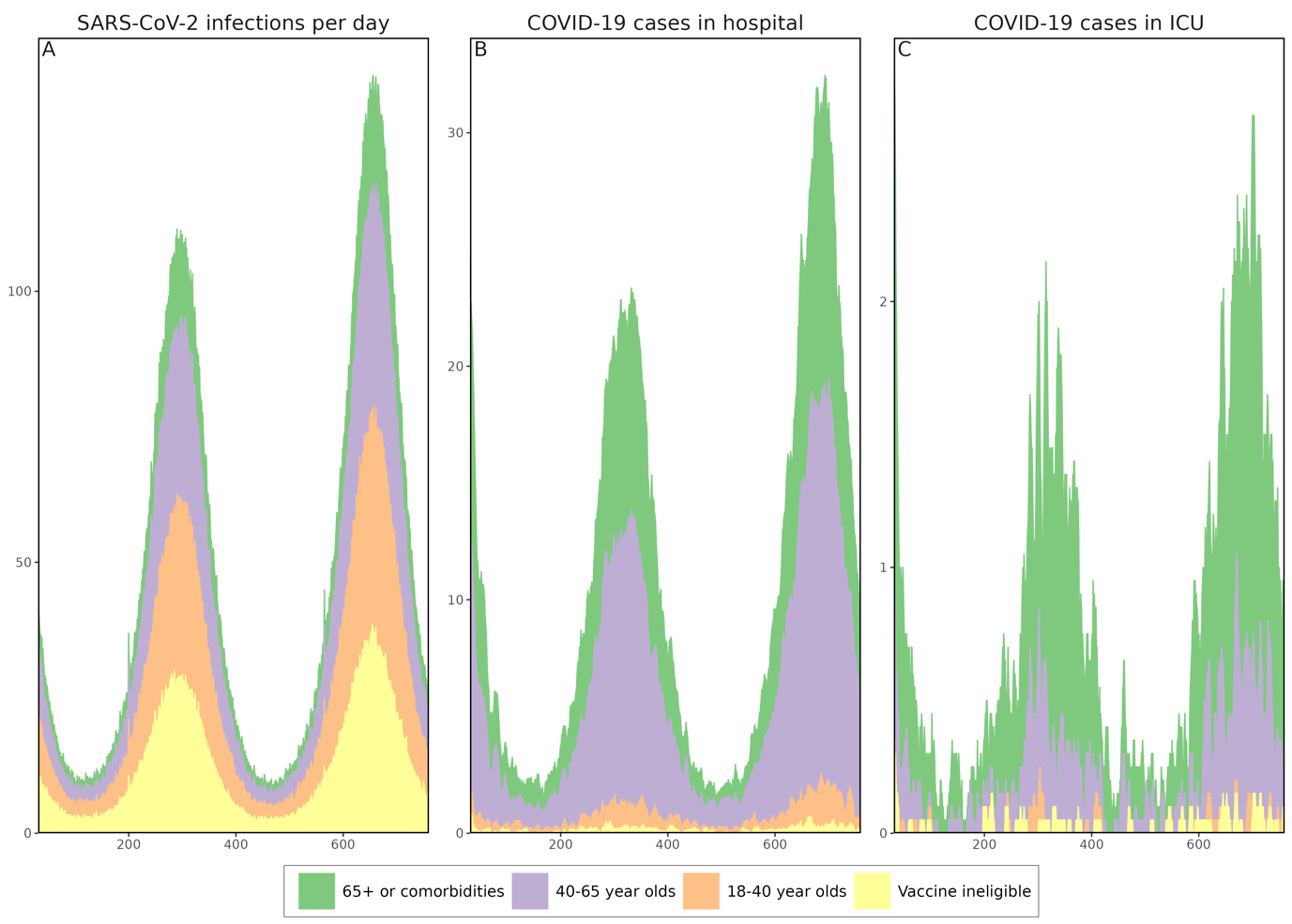


**Figure S10. Two-year temporal pattern for the “no treatment” scenario of Figure 1 showing the underlying age patterns for SARS-CoV-2 infections, COVID-19 cases in hospital, and COVID-19 cases in ICU for population of 100,000.** These age patterns highly depend on setting-specific demographics, vaccine coverage per age group, and past immunity within each age group. The uncertainty bounds represent 95% prediction intervals from 50 simulations per modelled scenario, capturing both parameter and stochastic uncertainty.

**
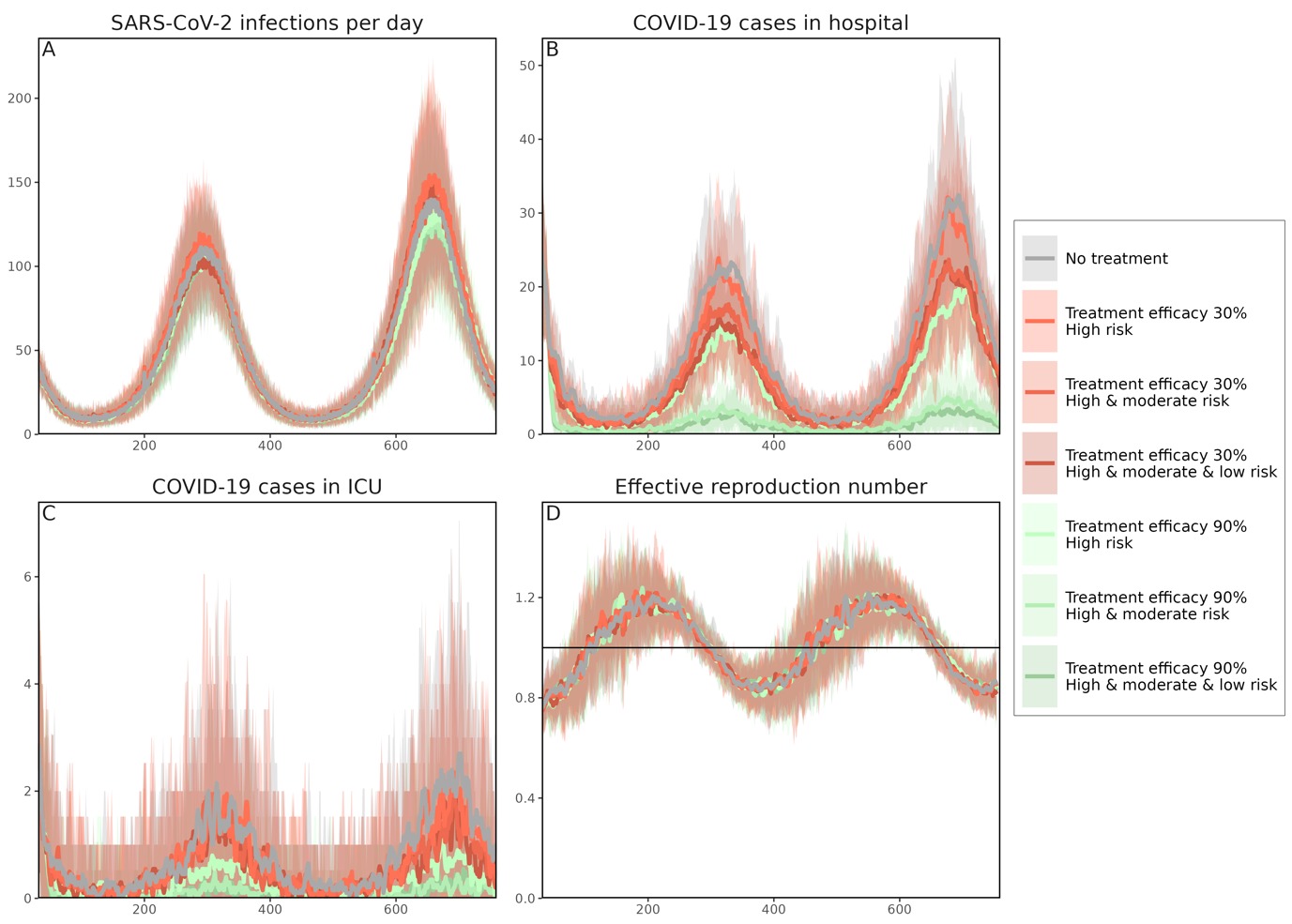
**

**Figure S11. Two-year temporal pattern for the seven scenarios of Figure 1, showing additional epidemiological SARS-CoV-2 outcomes for a simulated population of 100,000.** The waves reflect the seasonal pattern. In panels A, B, and C, the second wave is slightly higher than the first because of the annual arrival of novel emerging variants as illustrated in **Figure S10**. The uncertainty bounds represent 95% prediction intervals from 50 simulations per modelled scenario, capturing both parameter and stochastic uncertainty.


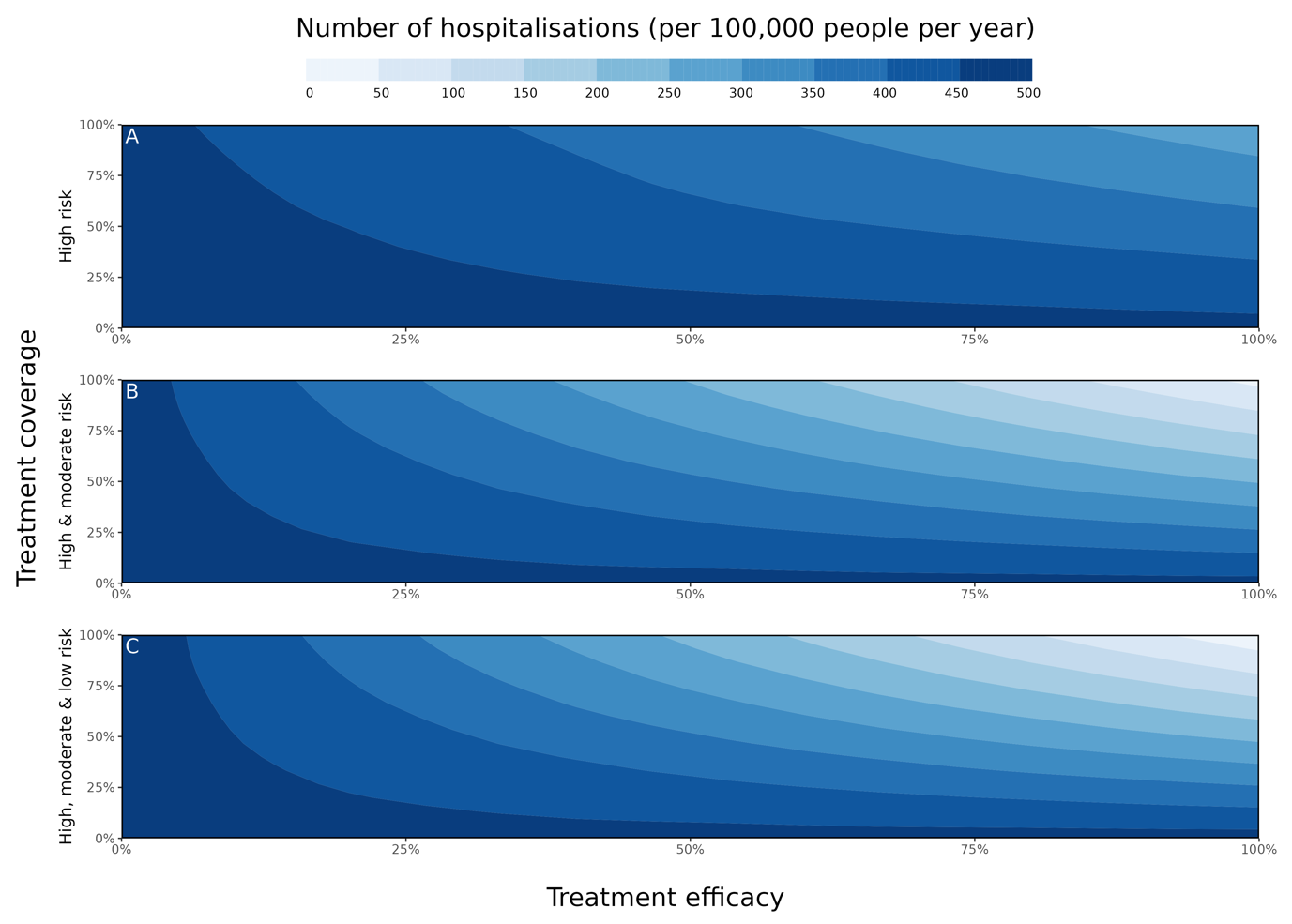


**Figure S12. Total number of hospitalisations per 100,000 people per year for the three target populations (y-axis, 0-100% treatment coverage per target population) and a full range of antiviral treatment efficacies (x-axis, 0-100% efficacy).**


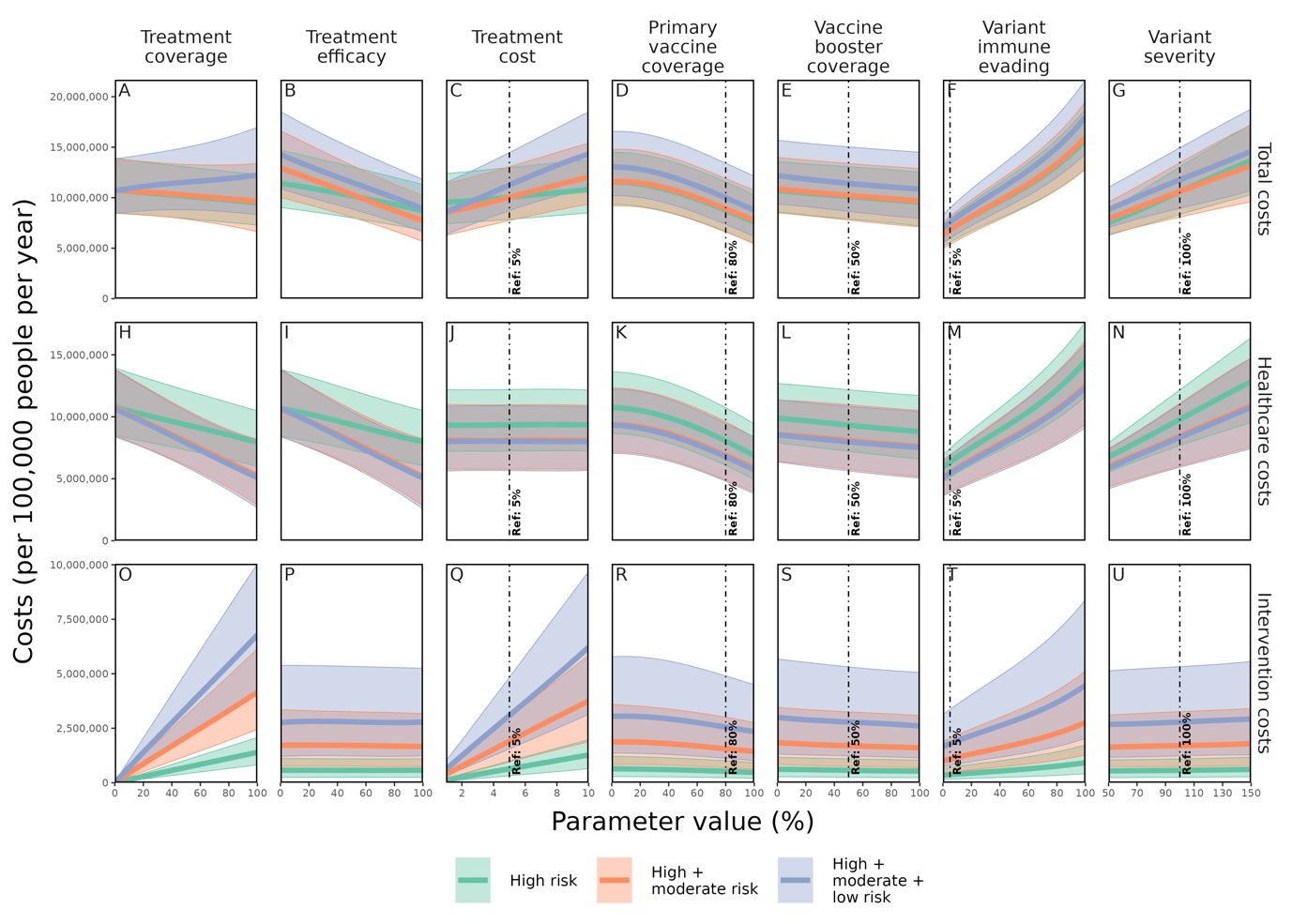


**Figure S13. Global sensitivity analysis for treatment coverage, treatment efficacy, treatment cost, primary and booster vaccination coverages, and variant immune-evading capacity and severity on intervention, healthcare and total costs.** The reference values and their ranges are presented in **Table 1**. The shaded regions present the range of model outcomes for each simulated parameter value whilst all other parameters are being varied across their plausible ranges.

**Interpretation of Figure S13**

The number of future hospitalisations and thus health costs is most sensitive to the immune-evading capacity of future variants, showing an exponential trend; the more immune-evading the future variants, the more cases, and thus more future hospitalisations and healthcare costs to be expected. The influence of the severity of future variants on the impact of treatments presents a similar but more linear trend. The total costs are a combination of the underlying healthcare costs as described above and the intervention costs, the latter evidently being most sensitive to the cost of treatment and treatment coverage. We stress here that whilst public health outcomes are highly sensitive to emerging variant properties, the cost-saving efficacy threshold as described in the main manuscript (**Figure 5**) is not particularly sensitive to such variant properties.

# Supplementary Table

**Table S1. Model parameters, their description and associated values and sources.** A schematic of the model structure and infection states is presented in **Figure S1**.

| Model parameter | Description | Value / distribution | Truncation bounds | Specifications and sources |
| --- | --- | --- | --- | --- |
| contacts | Number of contacts per person per  day, where a contact has a  transmission probability of beta | 9·8 – 10·5 | N/A | **Calibrated**  See human interaction section in SF2 |
| previously_infected | Conservative proportion of people infected in Europe since the start of the pandemic | 0·65 | N/A | ^16^ |
| beta | Transmission probability at maximum infectivty, susceptibility, and seasonality | 0·002 | N/A | See Infectiousness  per contact section in SF2 |
| seasonality_scaler | Multiplicative factor for effect of temperature on transmission  probability per contact | 0·3 | 0.2 – 0.8 | Calibrated in Shattock et al ^1^ |
| proportion_asymptomatic | Proportion of all cases that are asymptomatic | 0·33 | ? | ^17,18^ |
| presymptomatic | Number of days infectious before showing symptoms | 3·0 | Normal distribution, sd=1·0 | ^19^ |
| latency | Number of days in latency (infected but not infectious) state | 4·6 | Normal distribution,  sd=1·0 | ^20^ |
| infectious_mild | Number of days for which non-severe cases are infectious (excluding  presymptomatic phase) | 6·0 | Normal distribution, sd=1·0 | ^21^ |
| infectious_severe | Number of days for which severe cases are infectious (excluding presymptomatic phase) | 28·0 | Normal distribution, sd=1·0 | ^21^ |
| seek_hospital | Probability of seeking care if severe | 0·75 | N/A | Calibrated in Shattock et al ^1^ |
| onset_to_hospital | Number of days between symptom onset and hospitalisation | 13·0 | Normal distribution, sd=1·0 | Calibrated in Shattock et al ^1^ |
| diagnosis_delay | Delay between symptom onset and test/diagnosis | 1·0 |  | ^22^ |
| hospital_stay | Number of days a severe non-critical case spends in hospital before discharge | 9·0 | Normal distribution, sd=1·0 | ^23^ |
| hospital_to_icu | Number of days between hospital admission and ICU admission for cases that will become critical | 2·0 | Normal distribution, sd=1·0 | ^23^ |
| icu_stay | Number of days a critical case spends in ICU before transfer back to non-  ICU ward | 7·0 | Normal distribution, sd=1·0 | ^23^ |
| icu_stay_death | Number of days a critical case spends in ICU before death | 6·0 | Normal distribution, sd=1·0 | ^23^ |
| hospital_transfer | Number of days spent in non-ICU ward following discharge/transfer  from ICU | 2·0 | Normal distribution, sd=1·0 | ^24^ |
| home_death | Number of days between symptom onset and death for those not seeking  hospital care | 10·0 | Normal distribution, sd=1·0 | Assumed |
| death_critical_icu | Scale factors for death when critical in ICU | 1·0 | N/A | N/A |
| death_critical_non_icu | Scale factors for death when critical out of ICU | 3·0 | N/A | N/A |
| import_constant | Imported infections per day per 100,000 people | 1·5 | N/A | N/A |
| acquired_immunity | Immunity through natural infection | Median: 105 | Inverse logisitic regression,  slope 1·8 lb:0·15, ub:0·95 | ^25^ |
| vaccine_efficacy | Vaccine efficacy waning pattern. Number of days following vaccination (first dose) until full efficacy is attained | Median: First dose:  21 days,  Second dose: 70 days | Inverse logisitic regression,  slope: [1·8, 1·8]  lb: [0.00, 0.00]  ub: [0.40, 0.95] | ^25^ |
| Booster_efficacy | Booster efficacy waning pattern. Number of days following booster dose (third and subsequent doses) until full efficacy is attained. Booster administered 28 days after primary vaccine. | Median:  215 days | Inverse logisitic regression,  slope: 2·5  lb: 0·0  ub: 0·95 | ^12^ |

# Supplementary discussion

Besides the direct treatment costs, there are further financial factors that could influence the impact of antiviral treatments that were not considered here. While not a focus of this study they will be setting specific and include: associated non-targeted treatment costs (e.g., cost of labour of health care professionals, campaigning costs, shipping, insurance, waste), costs associated with a loss of workforce due to COVID-19 disease and hospitalisation, cost of illness (COI) at the societal level, national and patient willingness to pay, and opportunity costs. ^26,27^ Increased costs would generally lead to higher treatment efficacy requirements for a strategy to be cost-saving. In contrast, for scenarios where treatments lead to reduced costs (e.g. less loss of workforce), a lower treatment efficacy would be sufficient for a strategy to be cost-saving.

An additional model assumption to acknowledge is the assumption that once treated and recovered, individuals have similar properties to any other naturally recovered person. However, in reality, as treatment reduces the viral load compared to a non-treated infection, b-cell and t-cell responses are not as developed as they would be in a naturally recovered person. Therefore, we are likely slightly overestimating the treated individual’s immunity profiles and thus their protection against future exposure to the virus. We do however capture the delay between symptom onset, diagnosis, treatment application and the treatment taking effect, so this overestimation only applies to the later phase of the infection, making the difference relatively small.

# References

1 Shattock AJ, Le Rutte EA, Dünner RP, *et al.* Impact of vaccination and non-pharmaceutical interventions on SARS-CoV-2 dynamics in Switzerland. *Epidemics* 2022; **38**: 100535.

2 Le Rutte EA, Shattock AJ, Chitnis N, Kelly SL, Penny MA. Modelling the impact of Omicron and emerging variants on SARS-CoV-2 transmission and public health burden. *Communications Medicine* 2022; **2**: 93.

3 Kelly SL, Le Rutte EA, Richter M, Penny MA, Shattock AJ. COVID-19 Vaccine Booster Strategies in Light of Emerging Viral Variants: Frequency, Timing, and Target Groups. *Infect Dis Ther* 2022; published online Sept 12. DOI:10.1007/s40121-022-00683-z.

4 Shattock AJ, Penny MA. OpenCOVID git-repository: source-code. github.com/SwissTPH/OpenCOVID/tree/manuscript_treatment. .

5 Binois M, Gramacy RB, Ludkovski M. Practical Heteroscedastic Gaussian Process Modeling for Large Simulation Experiments. *Journal of Computational and Graphical Statistics* 2018; **27**: 808–21.

6 Binois M, Gramacy RB. hetGP: Heteroskedastic Gaussian Process Modeling and Sequential Design in R. *J Stat Softw* 2021; **98**. DOI:10.18637/jss.v098.i13.

7 Kerr CC, Dura-Bernal S, Smolinski TG, Chadderdon GL, Wilson DP. Optimization by Adaptive Stochastic Descent. *PLoS One* 2018; **13**: e0192944.

8 Swiss Federal Institute of Technology (ETHZ). COVID-19 Re. 2022; published online Dec 29.

9 Jones TC, Biele G, Mühlemann B, *et al.* Estimating infectiousness throughout SARS-CoV-2 infection course. *Science (1979)* 2021; **373**. DOI:10.1126/science.abi5273.

10 Kissler SM, Fauver JR, Mack C, *et al.* Viral dynamics of acute SARS-CoV-2 infection and applications to diagnostic and public health strategies. *PLoS Biol* 2021; **19**: e3001333.

11 Carabelli AM, Peacock TP, Thorne LG, *et al.* SARS-CoV-2 variant biology: immune escape, transmission and fitness. *Nat Rev Microbiol* 2023; published online Jan 18. DOI:10.1038/s41579-022-00841-7.

12 Andrews N, Stowe J, Kirsebom F, *et al.* Covid-19 Vaccine Effectiveness against the Omicron (B.1.1.529) Variant. *New England Journal of Medicine* 2022; **386**: 1532–46.

13 Swiss Federal Office of Public Health. COVID-⁠19 Switzerland. Information on the current situation: Vaccination. 2023; published online May 30. https://www.covid19.admin.ch/en/vaccination/status (accessed June 21, 2023).

14 Czernichow S, Bain SC, Capehorn M, *et al.* Costs of the COVID‐19 pandemic associated with obesity in Europe: A health‐care cost model. *Clin Obes* 2021; **11**. DOI:10.1111/cob.12442.

15 Czernichow S, Bain SC, Capehorn M, *et al.* Costs of the COVID‐19 pandemic associated with obesity in Europe: A health‐care cost model. *Clin Obes* 2021; **11**. DOI:10.1111/COB.12442.

16 European Commission. Speech by Commissioner Kyriakides at the Press Conference on COVID-19 – Sustaining EU Preparedness and Response: Looking ahead. Brussels, 2022.

17 Byambasuren O, Cardona M, Bell K, Clark J, McLaws M-L, Glasziou P. Estimating the extent of asymptomatic COVID-19 and its potential for community transmission: Systematic review and meta-analysis. *Official Journal of the Association of Medical Microbiology and Infectious Disease Canada* 2020; **5**: 223–34.

18 Nishiura H, Kobayashi T, Miyama T, *et al.* Estimation of the asymptomatic ratio of novel coronavirus infections (COVID-19). *International Journal of Infectious Diseases* 2020; **94**: 154–5.

19 Casey-Bryars M, Griffin J, McAloon C, *et al.* Presymptomatic transmission of SARS-CoV-2 infection: a secondary analysis using published data. *BMJ Open* 2021; **11**: e041240.

20 Wu Y, Kang L, Guo Z, Liu J, Liu M, Liang W. Incubation Period of COVID-19 Caused by Unique SARS-CoV-2 Strains. *JAMA Netw Open* 2022; **5**: e2228008.

21 Hakki S, Zhou J, Jonnerby J, *et al.* Onset and window of SARS-CoV-2 infectiousness and temporal correlation with symptom onset: a prospective, longitudinal, community cohort study. *Lancet Respir Med* 2022; **10**: 1061–73.

22 Kretzschmar ME, Rozhnova G, Bootsma MCJ, van Boven M, van de Wijgert JHHM, Bonten MJM. Impact of delays on effectiveness of contact tracing strategies for COVID-19: a modelling study. *Lancet Public Health* 2020; **5**: e452–9.

23 Rees EM, Nightingale ES, Jafari Y, *et al.* COVID-19 length of hospital stay: a systematic review and data synthesis. *BMC Med* 2020; **18**: 270.

24 European Centre for Disease Prevention and Control (ECDC). The European Surveillance System (TESSy) . Stokholm, 2020.

25 Spellberg B, Nielsen TB, Casadevall A. Antibodies, Immunity, and COVID-19. *JAMA Intern Med* 2021; **181**: 460.

26 Rajabi M, Rezaee M, Omranikhoo H, *et al.* Cost of Illness of COVID-19 and Its Consequences on Health and Economic System. *INQUIRY: The Journal of Health Care Organization, Provision, and Financing* 2022; **59**: 004695802211443.

27 Jeck J, Jakobs F, Kron A, Franz J, Cornely OA, Kron F. A cost of illness study of COVID-19 patients and retrospective modelling of potential cost savings when administering remdesivir during the pandemic “first wave” in a German tertiary care hospital. *Infection* 2022; **50**: 191–201.
